# Oxidomics of Human Plasma Reveals Lower Oxidized Lipids After Acute Intake of Polyphenol-Enriched Flaxseed Oil: A Randomized Crossover Exercise Trial

**DOI:** 10.1021/acs.jafc.6c08737

**Published:** 2026-09-22

**Authors:** Fawzan Sigma Aurum, Julia Brem, Matthias Strauss, Markus Gassner, Daniel König, Marc Pignitter

**Affiliations:** † Institute of Physiological Chemistry, Faculty of Chemistry, 27258 University of Vienna, Josef-Holaubek-Platz 2, Vienna 1090, Austria; ‡ Vienna Doctoral School in Chemistry (DoSChem), Faculty of Chemistry, University of Vienna, Währinger Str. 42, Vienna 1090, Austria; § Department of Nutrition, Faculty of Life Sciences, Section for Nutrition, Exercise and Health, 27258 University of Vienna, Vienna 1090, Austria; ∥ Centre for Sport Science and University Sports, Department of Sports Science, Section for Nutrition, Exercise and Health, University of Vienna, Auf der Schmelz 6, Vienna 1150, Austria; ⊥ Research Center for Food Technology and Processing, National Research and Innovation Agency, Yogyakarta 55861, Indonesia; # Vienna Doctoral School of Pharmaceutical, Nutritional and Sport Sciences, University of Vienna, Josef-Holaubek-Platz, Vienna 1090, Austria

**Keywords:** oxidized phospholipids, oxysterols, flaxseed oil, oxidative stress, high intensity interval training, lipidomics

## Abstract

Dietary antioxidants offer protection against oxidative bursts caused by high-intensity physical exercise. However, their acute effects on specific lipid oxidation products, especially oxidized phosphatidylcholine (oxPC) and oxysterols, remain underexplored in human plasma. Nineteen female participants underwent a crossover study involving pre-exercise intake of either polyphenol-enriched flaxseed oil (FO) or water (control), followed by standardized high-intensity interval training (HIIT). Plasma collected at four time points (pre- to post-HIIT) was analyzed via targeted LC-MS/MS for oxPCs and oxysterols and untargeted lipidomics using LC-QTOF-MS. FO significantly suppressed the postexercise rise in oxPCs (e.g., SONPC, KOOA-PC, KDdiA-PC) and nonenzymatically formed oxysterols (7-ketocholesterol, 7β-hydroxycholesterol). Orthogonal PLS-DA showed obvious postexercise group separation between FO-consuming subjects versus control. Untargeted lipidomics revealed broader remodeling of the phospholipidome in the FO group. This study provides novel evidence that polyphenol-rich FO acutely mitigates lipid peroxidation, supporting its potential as a nutritional strategy for oxidative stress management.

## Graphical abstract

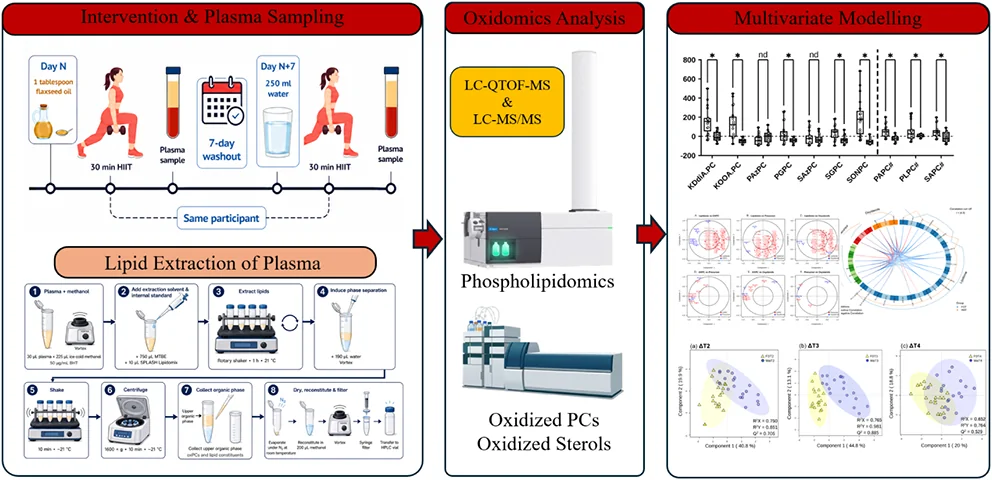

## 1 Introduction

Flaxseed or linseed, *Linum usitatissimum* (Linn.) oil (FO), renowned primarily for its abundant omega-3 alpha-linolenic acid (ALA) content, has drawn considerable scientific interest for its promising health-promoting effects, including anti-inflammatory, cardioprotective, and antioxidative properties.^1–3^ Despite these benefits, the inherent susceptibility of FO to oxidation due to the high unsaturation of ALA significantly limits its oxidative stability, thereby restricting its practical nutritional utility.^4^ Recognizing this limitation, recent innovations from our group have introduced polyphenol-enriched FO, resulting in an increase of oxidative stability by >30% compared to conventional FO.^5^ This advanced formulation holds considerable promise as a functional diet for counteracting oxidative stress and promoting metabolic health, yet a rigorous *in vivo* evaluation remains limited. Oxidative stress is characterized by an imbalance between reactive oxygen species (ROS) production and antioxidant defenses within the body and is widely recognized as a critical factor for the initiation and progression of numerous chronic diseases, including cardiovascular disorders,^6^ diabetes,^7^ neurodegenerative diseases,^8^ and inflammatory conditions.^9^ A major consequence of elevated oxidative stress is peroxidation of membrane lipids and cholesterol, leading to the generation of oxidized lipid species actively mediating inflammation, cellular injury, and vascular dysfunction.^10,11^

Within these oxidized lipid species, two classes have attracted special scientific interest due to their biological activities and clinical relevance: oxidized phospholipids (oxPLs)^12^ and oxysterols.^13^ Oxidized phosphatidylcholines (oxPCs), such as SONPC, PGPC, and KDdiA-PC, represent important subtypes of oxPLs known for their roles in triggering inflammatory signaling cascades, endothelial damage, and vascular dysfunction, thereby directly linking oxidative stress to the pathogenesis of cardiovascular diseases.^14–16^ Similarly, ROS-driven oxidation of cholesterol yields oxysterols, notably 7-ketocholesterol (7-KC) and 7β-hydroxycholesterol (7β-OHC), potent mediators implicated in mitochondrial damage, endothelial dysfunction, and atherosclerotic lesion development.^17–19^ Given their potent biological effects, quantifying these oxidized lipids provides valuable insight into the real-time oxidative status and the potential health benefits of antioxidant interventions.

One highly relevant model to study acute oxidative stress in humans is high-intensity interval training (HIIT).^20^ While moderate exercise elicits beneficial adaptive antioxidant responses, intense physical activity may increase ROS generation dramatically, leading to substantial oxidative stress.^21^ HIIT has thus emerged as an effective controlled experimental setting for testing the efficacy of dietary antioxidants in mitigating acute oxidative stress. Indeed, previous studies have demonstrated that dietary interventions rich in water-soluble antioxidants, such as polyphenol-containing berries^22^ and cocoa,^23^ can partially reduce exercise-induced oxidative markers. However, the role of polyphenol-enriched FO remains underexplored. By accumulating at lipid–water interfaces in lipoproteins and membranes, FO-borne antioxidants can quench peroxyl radicals, halt propagation, and chelate metals, plausibly limiting oxPCs/oxysterols and reshaping the PC profile during HIIT. Notably, antioxidant enrichment improved FO’s oxidative stability which prolonged the induction time by about 38% compared to conventional FO,^5^ motivating a test of its capacity to attenuate acute exercise-induced lipid oxidation *in vivo*.

Recent methodological advancements in lipidomics have revolutionized our ability to systematically profile oxidized lipid species with unprecedented sensitivity and specificity.^24–26^ While targeted lipidomics approaches enable the precise measurement of specific oxidized lipid biomarkers (e.g., oxPCs and oxysterols), untargeted lipidomics provides a comprehensive overview of changes across the broader lipidome. Integrating both targeted and untargeted lipidomics offers a powerful approach to understanding how dietary antioxidants influence complex lipid oxidation processes *in vivo*.^27^ Such integrative lipidomics can reveal not only how effectively an intervention suppresses known oxidized lipids but also how it reshapes the wider lipid metabolic landscape.

Despite this promising context, existing knowledge regarding the acute effects of polyphenol-rich FO supplementation on exercise-induced lipid oxidation remains sparse. Several studies have found that intervention of FO or isolated ALA supplementation has been linked to shifts in phytoprostanes, oxidation products of ALA, and oxylipins,^28–30^ but literature focusing on the exercise-induced oxidative stress has rarely targeted oxPC species or oxysterols, which more directly index the oxidation of membrane PCs and sterols. To our knowledge, this human trial study is the first to evaluate whether polyphenol-enriched FO can attenuate HIIT-provoked oxPC and oxysterol formation in healthy adults, contextualized by untargeted PC-focused lipidomics to capture plasma membrane remodeling. Therefore, this study aims to bridge an important knowledge gap, providing both mechanistic insights and potential biomarkers of oxidative stress.

Addressing this gap, we designed a controlled, randomized crossover human study to test the hypothesis that acute supplementation with polyphenol-enriched FOs could effectively reduce exercise-induced lipid oxidation. We utilized both targeted and untargeted lipidomics to provide comprehensive insights into changes in oxPCs, oxysterols, and the overall lipidome profile.

The primary aim of this research was to determine whether preexercise supplementation with polyphenol-enriched FO can acutely mitigate exercise-induced oxidative lipid damage, specifically assessing changes in oxPCs and oxysterols in human plasma. Furthermore, by exploring broader lipidomic responses to antioxidant intervention via an untargeted lipidomics approach, this study aimed to provide a holistic understanding of lipid metabolism under oxidative stress.

## 2 Materials and Methods

### 2.1 Materials

#### 2.1.1 Samples

The present study was conducted following the design of a previously published study, involving 19 healthy female participants aged between 18 and 40 years with a BMI ranging from 18.5 to 25.0 kg/m^2^. The study followed a crossover design, where each participant consumed both the optimized FO and water, as a control, on separate experimental days, with a washout period of 7 days between interventions to minimize carryover effects. As the present study forms part of a larger research program, participant selection criteria and the CONSORT flow diagram have been reported elsewhere.^31^

Participants were instructed to refrain from exercise, alcohol consumption, and any significant dietary changes the day before each test. Additionally, the participants maintained dietary logs throughout the study to ensure consistency in their food intake. Ethical approval for the study was obtained from the University of Vienna, with the approval number 00743-1.12.2021. Written informed consent was obtained from all participants prior to their inclusion in the study. The subjects were then admitted to an experimental design setup for a HIIT as illustrated in Figure 1. On the day of the experiment, the participants were required to fast for a minimum of 12 h prior to the commencement of the study. On the morning of the experiment, the participants had their blood drawn for the first time 30 min after their arrival (T1). Participants then consumed, within 10 min, either 5 g (one tablespoon) of the polyphenol-enriched flaxseed oil, immediately followed by 250 mL of water (FO arm), or 250 mL of water alone (control arm). The identical water volume administered in both arms ensured matched hydration status and gastric volume. This is to ensure adequate hydration and optimized conditions for both study arms to complete the exercise protocol safely. After resting for a further 2 h, blood was drawn (T2), then HIIT was performed for a duration of 30 min, immediately after which blood was drawn once more (T3). After further 15 min, blood was drawn for the fourth and final time (T4). Whole blood was collected directly from the participants in tubes containing ethylenediaminetetraacetate (EDTA) (Greiner Bio-One GmbH, Kremsmünster, Austria) to prevent clotting and subsequently centrifuged at 1130 × g for 15 min at 4 °C (Centrifuge 5415 R, Eppendorf, Hamburg, Germany). The plasma samples were stored at −80 *°*C until further analysis. Prior to sample preparation, the samples were thawed for 40–45 min on ice and vortexed.

**Figure 1:**
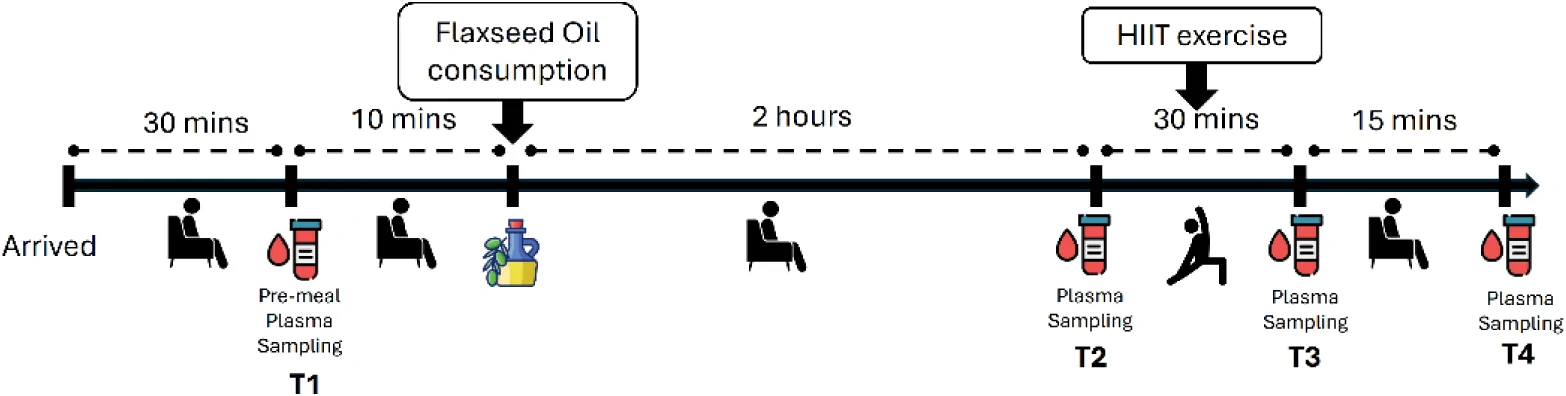
Study design with plasma collection at different time points (T1, T2, T3, and T4).

The optimized FO used in this study was obtained from OFS Lipid Legends GmbH, Vienna, Austria. This FO polyphenolic content of this is more than 8.22 ± 0.08 mg GAE/100 g oil which is 12-fold higher than in nonoptimized FO (details in Table S6).^5^

#### 2.1.2 Chemical Reagents

The chemical reagents used in this study were of LCMS grade. Lipid internal standards (IS) used in this study were purchased from Avanti Polar Lipids, LLC (Alabama, USA) including SPLASH LIPIDOMIX Mass Spec Standard {(15:0–18:1­(d7) PC, 15:0–18:1­(d7) PE, 15:0–18.1­(d7) PG, 15:0–18:1­(d7) PI, 15:0–18:1­(d7) PS, 15:0–18:1­(d7) PA, 18:1­(d7) Lyso PC, 18:1­(d7) Lyso PE, 18:1­(d7)-chol ester, 18:1­(d7) MG, 15:0–18:1­(d7) DG, 15:0–18:1­(d7)-15:0 TG, d18:1–18:1­(d9) SM, and Cholesterol (d7)}, 1-palmitoyl-2-glutaryl-*sn*-glycero-3-phosphatidylcholine (PGPC) 7-ketocholesterol-d7, 7β-hydroxycholesterol-d7, 25-hydroxycholesterol-d6, 7-ketocholesterol, 7β-hydroxycholesterol, 25-hydroxycholesterol. Other reagents are acetone (Carl Roth GmbH + Co. KG, Karlsruhe, Germany), acetonitrile (VWR International, Radnor, PA, USA), ammonium acetate (Sigma-Aldrich, St. Louis, MO, USA), butylated hydroxytoluene (Thermo Fisher Scientific, Waltham, MA, USA), formic acid (VWR International, Radnor, PA, USA), isopropanol (Carl Roth GmbH + Co. KG, Karlsruhe, Germany), methanol (Merck KGaA, Darmstadt, Germany), methyl *tert*-butyl ether (Merck KGaA, Darmstadt, Germany), and LCMS grade water (VWR International, Radnor, PA, USA).

### 2.2 Lipid Extraction for Oxidized Phospholipids and Untargeted Lipidomics

The extraction of lipid compounds from plasma samples was performed using an MTBE protocol, based on previously published methods.^32,33^ Initially, human plasma samples were thawed on ice for approximately 40–45 min and then homogenized by vortexing. Subsequently, 30 μL of plasma was combined with 225 μL of ice-cold methanol containing 50 μg/mL butylated hydroxytoluene (BHT), followed by vortexing. To this mixture, 750 μL of MTBE was added, along with 10 μL of a 1:10 dilution of SPLASH Lipidomix IS in MeOH.

The mixture was subjected to continuous agitation at 21 °C for 1 h using a rotary shaker. Thereafter, 190 μL of water was added to induce phase separation, and the mixture was vortexed again. Following an additional 10 min of shaking, samples were centrifuged at 1600 × g for 10 min at ∼21 °C. The upper organic phase, containing the oxPCs and lipid constituent, was carefully collected and transferred into a Falcon tube.

The collected organic extract was evaporated to dryness under a nitrogen stream at room temperature. The resulting residue was reconstituted in 200 μL of methanol, vortexed thoroughly, and filtered using a 0.20 μm polyvinylidene fluoride (PVDF) syringe filter (15 mm diameter). The filtrate was transferred to a high-performance liquid chromatography (HPLC) vial equipped with an insert. A pooled quality control (QC) sample was prepared by combining aliquots from all individual samples.

### 2.3 Lipid Extraction for Oxidized Sterols

The extraction of free oxidized sterols from human plasma was performed according to the protocol described by Helmschrodt et al.,^34^ with slight modifications. Briefly, 100 μL of thawed plasma was mixed with 400 μL of ice-cold methanol containing 50 μg/mL BHT to minimize autoxidation and spiked with 10 μL of an IS mix containing 7-KC-d7, 7β-OHC-d7, and 25-OHC-d6. The mixture was vortexed and incubated on ice for 10 min to facilitate protein precipitation. After centrifugation at 14,000 × g for 10 min at 4 °C, the supernatant was transferred into a clean glass tube and evaporated to dryness under a nitrogen stream at room temperature.

The dried residue was reconstituted in 100 μL of methanol:water (80:20, v/v), vortexed, and passed through a 0.20 μm PVDF syringe filter prior to transfer into HPLC vials with inserts. Samples were stored at −80 °C until LC-MS/MS analysis.

### 2.4 OxPC and Oxysterol Lipid Profiling Using LC-MS/MS

PCs, their oxidized derivatives oxPCs, and oxysterols were analyzed using liquid chromatography-tandem mass spectrometry (LC-MS/MS). The LC system included a binary pump (LC-20AD) and an autosampler (SIL-20AC) from Shimadzu (Kyoto, Japan). Chromatographic separation was performed on a Phenomenex Kinetex C18 column (2.6 μm, 100 Å, 150 × 2.1 mm), maintained at 40 °C. An injection volume of 30 μL of the reconstituted sample was used. Mobile phase A consisted of acetonitrile and water (60:40, v/v), and mobile phase B was a mixture of acetonitrile and isopropanol (20:80, v/v), each supplemented with 0.1% formic acid and 10 mM ammonium acetate. The gradient elution began at 40% B, increased linearly to 100% B by 15 min, held for 10 min, and then returned to 40% B within 2 min. A subsequent 10 min re-equilibration step resulted in a total run time of 37 min, with a flow rate set at 0.3 mL/min.^32^

The mass spectrometer (LCMS-8040, Shimadzu, Kyoto, Japan) was operated in polarity-switching mode, alternating between positive and negative ionization during the chromatographic run. This dual-mode acquisition enabled the comprehensive detection of lipid species with varying ionization efficiencies.

As reported in previous studies,^15,35^ the measurement of PC and oxPC species was based on a single MRM transition per analyte. The diagnostic fragment ion at *m*/*z* 184, derived from the phosphocholine headgroup, was employed as the marker transition because of its high specificity for both PCs and oxPCs. In line with these approaches, this study applied a one-transition-per-compound strategy. Table S1 (Supplementary) lists the MRM transitions used for precursor PCs and oxPCs, respectively, while Table S2 provides the transitions and collision energies for the IS. Table S3 and S4 list the MRM transitions of oxysterols and their authentic standards, respectively.

The analytical sequence was as follows: initiated with several blanks, the QC sample was measured twice, and after that, the QC was measured every tenth injection in between the samples, and they were bracketed with blanks. In total, 249 injections were conducted, of which 28 were QC injections. Prior to and following each QC injection, a blank was measured to remove potential carryover from previous injections. The standard series was measured at the beginning and at the end of injections to ensure analytical repeatability. Further methodological details for this analysis are provided in Supporting Information 2 to ensure transparency, in accordance with the lipidomics minimal reporting checklist.^36^

### 2.5 Untargeted Phospholipidomics Using LC-Q-TOF-MS

A liquid chromatography system (LC-20AD with SIL 20ACHT autosampler, Shimadzu, Kyoto, Japan) coupled to a high-resolution Quadrupole-Time of Flight-MS (Agilent 6546, Agilent, Singapore) was utilized to analyze the lipid phase of the plasma sample in untargeted mode following Cajka et al.,^37^ with slight modification. The separation of the lipid analytes was performed using a Zorbax Eclipse Plus C18 (Agilent, USA) column with a diameter of 2.1 × 50 mm and a particle size of 1.8 μm. This column oven was maintained at 60 °C throughout the measurement, for 14.5 min. The mobile phases used were identical to the previous oxPC measurement. The flow rate was set to 0.6 mL/min, and the starting gradient was at 5% B. The gradient was maintained for 1 min and then increased for 4 min to 95% B. Over the next 7.5 min, it increased to 100% B, and over the next min, it decreased again to 5% B and the re-equilibration time was another 2 min. The injection volume of the sample was 5 μL.

Reference ions with *m*/*z* 121.0509 (C_5_H_4_N_4_) and *m*/*z* 922.0098 (C_18_H_18_O_6_N_3_P_3_F_24_) in ESI positive ion mode were used. These reference compounds were continuously pumped into the Q-TOF system at a flow rate of 15 μL/min. The Q-TOF was run in positive ion mode, low mass range of *m*/*z* 50–1700, in the acquisition mode of Auto MS/MS with maximum 4 scans per cycle and an acquisition time of 250 ms per spectrum. This was the case for both the precursor and the product ion. The precursor target was set in the range of *m*/*z* 50–1500 for MS1 and the product ion ranging from *m*/*z* 50–1000 for MS2. Precursor absolute threshold was 25000 counts, while the relative threshold was 0.01%. The CE was set to 20 and 50 eV. The data were stored in both centroid and profile formats. In details some of the Q-TOF parameters were as follows: gas temp. 300 °C, drying gas 3.0 L/min, nebulizer 15 psi, VCAP 3500 V and 0.063 μA, chamber 0.31 μA, nozzle voltage 2000 V, sheath gas temp. 125 °C, sheath gas flow 3.0 L/min.

More detailed transparency about the methodology used for this analysis is provided in Supporting Information 3, according to the lipidomics minimal reporting checklist.^36^

### 2.6 Data Preprocessing

#### 2.6.1 OxPC Data Preprocessing

The oxPC data preprocessing was carried out in RStudio using our R in-house script (ver. R-4.3.1) (https://github.com/ozanfaw/QC_normalization/blob/main/Skyline_QC_LOWESS_RSD.R). The first step involved preprocessing the raw data using the QC for normalization.^60^ This was achieved by plotting the QC curve using locally weighted scatterplot smoothing (LOWESS). In general, LOWESS creates a smooth trend line through scattered data points by fitting small linear regressions to localized subsets of the data, LOWESS smoothing was performed using the lowess­() function in R (stats package), with a span of 0.5, linear local fitting (degree = 1), and two robust iterations to reduce the effect of outliers. By applying this procedure, the impact of interbatch variability and instrument drift on the analytical results could be reduced, thereby enhancing overall data quality and reliability.^38^ The data set was filtered to remove features with a standard deviation (SD) *>*30%. A linear model was then created. The mean value of the QC was divided by each QC value, and then it was multiplied by all sample values for the normalization. After that, the samples were multiplied by the average value of the IS and then divided by the respective IS value of the respective sample. After the removal of outliers using the IQR, the normalization process was complete. Following the normalization process, time point 1 was selected as the baseline. Then, time points 2, 3, and 4 were subtracted from those of time point 1.

#### 2.6.2 Oxysterol Data Preprocessing

The data preprocessing of oxysterols (25-OHC, 7β-OHC, and 7-KC) followed a similar method as described for the oxPC in the previous section with slight adjustment. In brief, preprocessing steps for the samples began with the selection of the highest peak area of the MRM transitions for qualification and quantification for each analyte. This was achieved by taking the highest average of peak areas from time point 3, as this was expected to yield the highest peak areas. The data set was examined for outliers based on the IQR, which were found to follow a normal distribution. After that, the normalization with the IS was initiated. For oxysterol quantification, linear calibration curves (R^2^ > 0.990) were generated using external standards across a concentration range of 0.5–100 μg/mL. Instrument performance was monitored by assessing the consistency of IS signals and retention times across all samples and batch, in this experiment, no significant drift or variability was observed during the analysis.

#### 2.6.3 Untargeted Lipidomics Data Preprocessing

The lipidomics raw data were converted from the agilent .d format into.abf using Reifycs Abf converter. Specific measurement parameters for the lipidomics analysis were established in MS-DIAL (Systemomicslab, Japan; version 5.2.240424.3):^39^ positive ion mode was selected as the ionization method, and the target omics was specified as lipidomics. Collision type was set to both CID (collision-induced dissociation) with data type settings for MS1 and for MS/MS as centroid data. The mass range for MS1 was defined as 50 to 1500 Da, while the accurate mass tolerance thresholds were set at 0.01 Da for MS1 and 0.05 Da for MS2, ensuring the precision in lipid identification. The database for identifying the lipid compounds was taken from MS-DIAL LipidBlast Fork and GNPS open-source databases. The threshold level of identification was set to 80% similarity.

The identification protocol of the detected compounds follows the Metabolomics Standard Initiative.^40^ Following the initial processing steps, the data set underwent further refinement through normalization and quality checks. The lipid compounds detected were normalized to the IS splash lipidomics (Table S1) and QC, which accounted for variability across samples. Duplicate detected features were removed, and the data set was filtered to focus on a specific PC class as identified by the available database, ensuring that only relevant data were analyzed. Outliers were detected through the IQR method, and any identified outliers were excluded to maintain the integrity of the data set. Features were filtered based on quality control metrics (i.e., RSD of <30%). The final data set was curated by removing compounds that did not exhibit an MS2 signal, resulting in a significant reduction of initially detected peaks from over 70,000 to approximately 130 solid and well-curated lipids in the class of phospholipids.

### 2.7 Multivariate Analysis

Orthogonal Partial Least Squares–Discriminant Analysis (OPLS-DA), a supervised multivariate method, was applied to the data sets derived from both targeted and untargeted lipidomic analyses. Data preprocessing as mentioned earlier included normalization using IS and QC-based LOWESS signal correction, followed by autoscaling. The modeling was performed using the MetaboAnalystR 3.0 R-Package,^41^ employing 10-fold cross-validation to assess the robustness and predictive performance of the model and to avoid overfitting. A total of 10 latent variables were retained, and 1000 permutation tests were executed to validate the model. Variables with a Variable Importance in Projection (VIP) score >1.0 and p-value <0.05 were considered as major contributors to class separation.^38^

This approach was employed across the data sets of oxPCs and untargeted phospholipids funneled from the untargeted lipidomics, particularly PCs and ether-linked PC-Os. Time-dependent comparisons were made across different postintervention time points (T2, T3, and T4), relative to the fasting baseline (T1). The output of the OPLS-DA models was visualized using 2-D score plots and VIP bar plots, enabling the identification of discriminatory lipid species influenced by the consumption of modified FO versus water.

Additionally, unsupervised hierarchical clustering analysis was also visualized using MetaboAnalystR 3.0 to identify natural grouping patterns and evaluate overall lipidomic similarity across groups. The Euclidean distance metric with Ward’s linkage was used for clustering, and the results were visualized as heatmaps for the top discriminant features. To integrate the three complementary data blocks collected at the immediate postexercise time-point (T3): (i) untargeted PC lipidomics, (ii) targeted oxPCs, and (iii) targeted oxysterols, we carried out pairwise sparse PLS correlation analysis (mixOmics, n = 38 variables; κ = 0.8). Spearman coefficient at α = 0.05 in a two-tailed test is approximately 0.46. A cutoff of |r| > 0.50 therefore retains only associations that exceed nominal significance for the sample size of this study.

### 2.8 Multivariate Integration of the Data Set

Integration of the four lipid data blocks was performed in R (version 4.3.1) (R Core Team) using the mixOmics package (version 6.30.0). The blocks comprised the untargeted phospholipidome, the targeted oxPCs, the precursor PCs, and the oxysterols, following outlier removal based on the IQR and data cleaning. Samples were matched across the four blocks by sample identifier, and the response factor was defined with two levels: FOT and WAT.

Pairwise relationships between the blocks were modeled by partial least-squares regression in canonical mode. Each block was mean-centered and scaled to unit variance prior to modeling, and each of the six block pairs was fitted with two components. Feature loadings were displayed as correlation circle plots with a correlation cutoff of 0.5, in which the radial projection of a variable expresses its correlation with the two model components.

Cross-block integration was carried out by multiblock sparse PLS-DA (DIABLO) using two components and a full design matrix, so that every block pair was connected with equal weight. The number of variables retained per block and per component was tuned over an adaptive grid, in which the candidate values for each block were scaled to the number of features in that block, using 5-fold cross-validation repeated 10 times, with the random seed fixed at 456. The tuned values were [[keepX]] variables for the phospholipidome, oxPC, precursor PC, and oxysterol blocks, respectively. The final model was fitted with the full design and the tuned sparsity, and was visualized as a circos plot displaying correlations of |r| ≥ 0.5 between the selected features, with the group mean of each feature shown on the outer track.

### 2.9 Statistical Analysis

All other statistical analyses were conducted using GraphPad Prism Version 10.0R. Normalized data from both targeted (oxPCs, oxysterols) and untargeted (lipidomic) analyses were subjected to appropriate statistical testing based on data distribution and experimental design. For comparisons between paired conditions (e.g., water vs FO within the same individuals at corresponding time points), paired two-tailed *t* tests were applied when normal distribution was assumed. In cases where normality was not established, the nonparametric Wilcoxon signed-rank test was used. Multiple comparisons across compounds were corrected using the Holm–Sidak method to control for false discovery rate. A significance threshold of α = 0.05 was adopted throughout. Statistically significant differences were annotated using asterisk notation, with *p* < 0.05 considered significant.

## 3 Results and Discussion

### 3.1 Suppression of Exercise-Induced Oxidative Stress with Antioxidant-Rich Flaxseed Oil

The main aim of this study was to investigate whether consumption of a modified, polyphenol-enriched FO could suppress the rise in oxidative lipid biomarkers induced by high-intensity physical exercise. Specifically, the study focused on two key classes of lipid oxidation products in human plasma samples, i.e., oxPCs and oxysterols. Additionally, untargeted lipidomics was employed to capture broader phospholipidomic dynamics resulting from exercise and dietary intervention. As a result of the exclusion of plotted QC curves containing a SD greater than 30%, some compounds, specifically HOOA-PC, PONPC, POVPC and SLPC, were excluded from further evaluation, an approach aligned with current QC guidance in untargeted LC–MS metabolomics.^42^ To exemplify a compound with an acceptable RSD, Figure S1 shows the plotted QC curve of the compound KDdiA-PC and Figure S2 shows the compound which is removed due to QC selection.

Phospholipids which contain polyunsaturated fatty acids at the sn-2 position are the primary targets for ROS in cell membranes.^43^ When oxidized, they form biologically active derivatives such as KDdiA-PC, KOOA-PC, PGPC, SGPC, and SONPC, which are implicated in endothelial dysfunction and inflammation.^14^ In this study, after ingestion of FO, participants showed a significant suppression of oxPCs, even before exercise commenced (ΔT2), indicating a rapid systemic antioxidant effect (Figure 2a). Notably, KDdiA-PC and KOOA-PC decreased by 31.3% and 60.9%, respectively, relative to baseline (T1). By contrast, SGPC paradoxically increased, suggesting that not all oxPC species were uniformly regulated which is consistent with the known species-specific and sometimes opposing biological activities of distinct oxPCs.^44^

**Figure 2:**
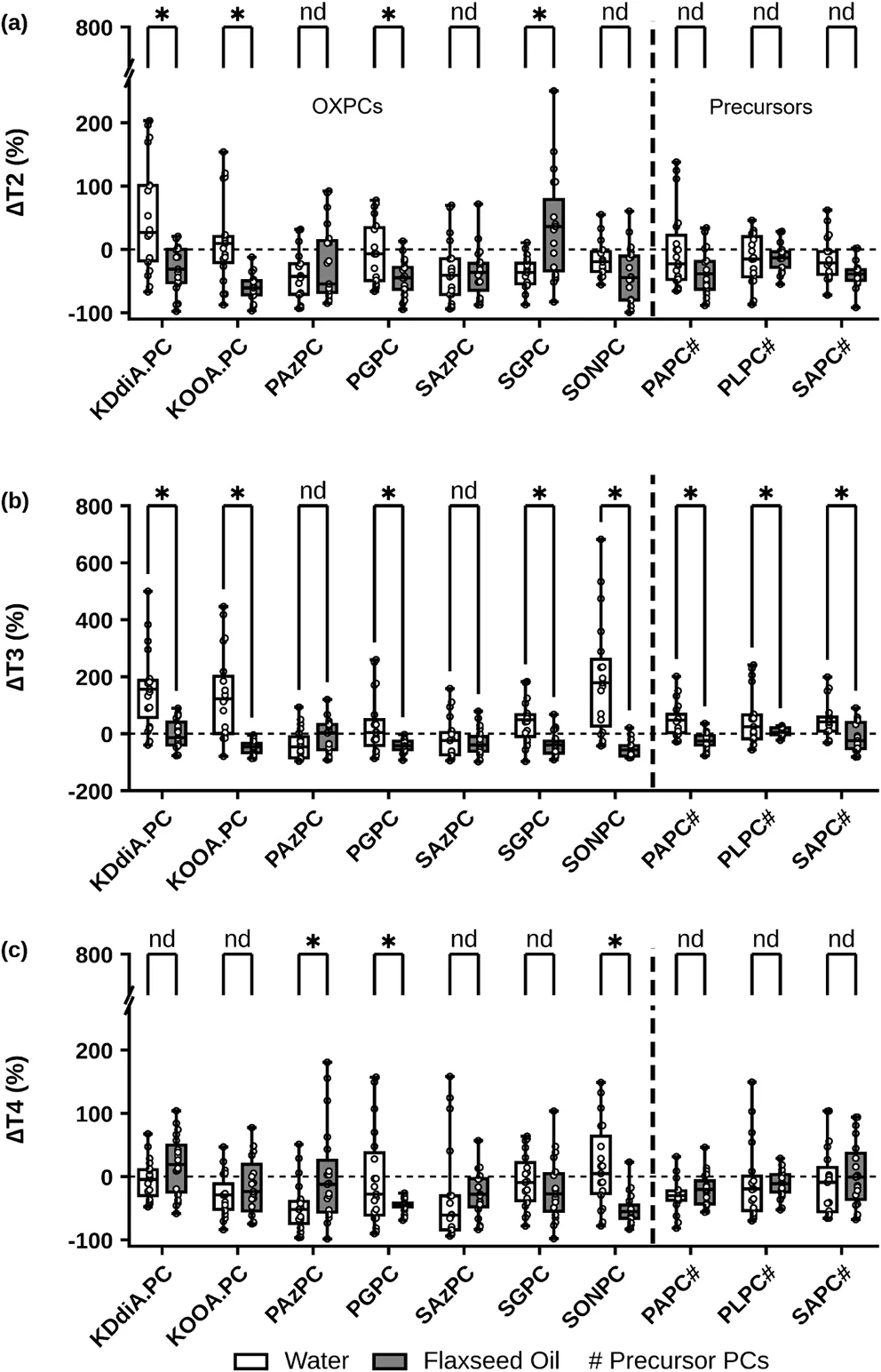
oxPC and PCs for water and FO groups at different time points [a. ΔT2(%); b. ΔT3(%); c. ΔT4(%)] relative to baseline (T1). Data are presented as mean ± SD (*n* = 19); asterisk (*) indicates significant difference (p *<* 0.05) between both groups determined by using paired multiple *t* test and Holm-Sidak’s multiple comparison test.

Following exercise (ΔT3) (Figure 2b), the control group exhibited dramatic increases in five of the seven quantified oxPC species, namely KDdiA-PC, KOOA-PC, PGPC, SGPC, and SONPC, with the largest changes observed for SONPC and KDdiA-PC surged by over 150–190%. The azelaoyl species PAzPC and SAzPC did not increase significantly and showed median changes at or below baseline in both arms. HIIT therefore triggered a species-selective rather than a uniform increase across the oxPC panel. The direction of these changes is consistent with the exercise literature documenting rises in lipid peroxidation markers after intense bouts.^45,46^ However, participants pretreated with FO showed no such increases, and in fact, many oxPC levels declined relative to baseline, strongly suggesting that FO attenuated the ROS surge. By 15 min postexercise (ΔT4), a partial normalization of oxPCs occurred in both groups, yet the FO group retained significantly lower levels of PGPC and SONPC (Figure 2c). The observed dynamics suggest that oxPCs are rapidly reactive intermediates, and their persistence postexercise may be limited by enzymatic degradation, incorporation into lipoproteins, or further oxidation.^47^ Notably, the precursor species PAPC, PLPC, and SAPC also differed between arms following exercise. At ΔT3, all three were significantly lower in the FO arm than in the water arm (Figure 2b), reflecting an increase relative to baseline in the water arm while concentrations in the FO arm remained close to baseline. No significant between-arm differences were present for these species before exercise (ΔT2, Figure 2a).

Furthermore, using the oxPCs as the features, the OPLS-DA score plots confirmed these trends (Figure 3). At ΔT3, group separation between the FO and water-consuming individual plasma samples is clear (Figure 3b), with VIP scores identifying SONPC, KOOA-PC, KDdiA-PC, and SGPC as the discriminatory features (Figure S3). These findings also reinforce the idea that oxPCs may serve as indicators of acute oxidative stress, and their suppression by dietary antioxidants can be robustly monitored by mass spectrometry.

**Figure 3:**
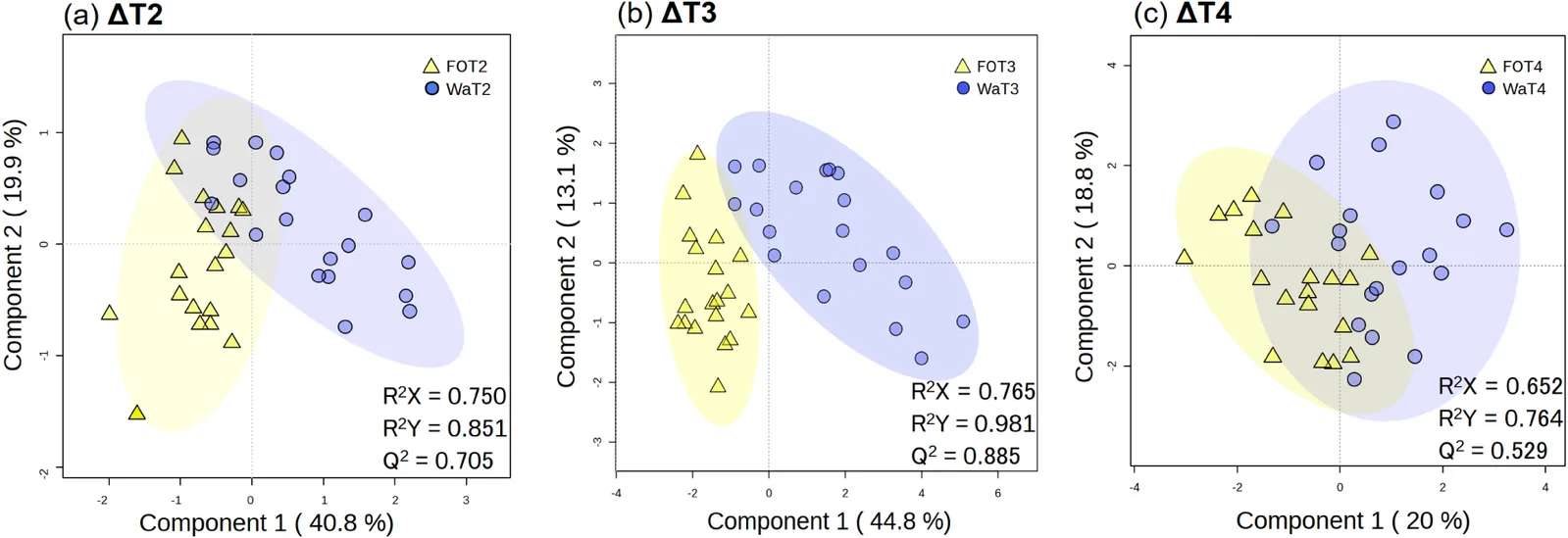
Multivariate analysis (OPLS-DA) using the oxPC and PCs as features for comparison between (a) ΔT2 (T2–T1), (b) ΔT3 (T3–T1), and (c) ΔT4 (T4–T1) with the baseline of T1 for the individuals who consumed water (WaT) vs FO (FOT).

The OPLS-DA score plots (Figure 3 a–c) provide a concise multivariate synopsis of how the oxPC profile evolves across the nutritional-exercise time course. From a multivariate standpoint, the ΔT2 model (40.8% + 19.9% total explained variance) resolves the water and FO clusters along the predictive latent variable, indicating that the polyphenol-enriched oil modified the plasma oxPC space within 2 h of ingestion. However, the 95% Hotelling ellipses still overlap appreciably, although this ellipse statistically describes as confidence regions of the OPLS-DA, but it also may suggest that interindividual biochemical noise partially masks the nutritional effect. Immediately postexercise (ΔT3) the predictive power of component 1 increases to 44.8%, and the orthogonal latent variable (Component 2 = 13.1%) tightens both ellipses (Figure 3b). This pattern is typical of a supervised model in which the treatment effect (FO vs water) amplifies under oxidative challenge, while orthogonal filtering removes within-group covariance; in other words, exercise unmasks the antioxidant advantage of FO by driving the control group (water) to a more pro-oxidant lipidome.

After, 15 min recovery (ΔT4), the group centroids remain separated (Figure 3c), yet component 1 now explains only 20% of variance, reflecting a rapid metabolic convergence, or their metabolisms quickly aligned again once exercise-induced ROS production subsides. Taken together, the shifting group centers and shrinking or inflating ellipses show how rapidly lipid oxidation changed and demonstrate that OPLS-DA can clearly separate the treatment effects from time effects in complex LC–MS data. The intervention delivers ALA together with phenolic antioxidants enriched in FO. These phenolics may not only scavenge radicals but also recycle lipid-phase tocopherols, thereby curbing propagation of PUFA-derived lipid-peroxyl radicals and throttling formation of truncated aldehydic PCs at ΔT3.

Although oxPCs in human plasma have not been studied immediately postexercise, elevated levels are commonly linked to obesity and age-related diseases, both associated with oxidative stress and cardiovascular risk. Obese individuals showed reduced plasma PCs,^48^ implying a shift toward oxPC formation. Truncated oxPCs such as POVPC, SOVPC, PONPC, and SONPC were elevated in myocardial infarction and renal ischemia/reperfusion patients,^15,49^ aligning with the present findings where SONPC showed the highest postexercise increase:

### 3.2 Sterol Oxidation as a Complementary Stress Signal

Whereas oxPCs reflect membrane lipid oxidation, oxysterols represent oxidative modifications of cholesterol, providing a sterol-specific perspective on lipid peroxidation. Among them, 7β-OHC and 7-KC are nonenzymatic, while 25-OHC can be formed both enzymatically and nonenzymatically.^50^ ROS-derived oxysterols are associated with mitochondrial dysfunction, endothelial injury, and foam cell formation.^51^ As illustrated in the boxplot of Figure 4a, at the peak of oxidative stress (T3), the average level of 7β-OHC in the control group reached 84.9 μg/mL, a striking elevation compared to only 5.4 μg/mL in the FO group. And 7-KC followed a similar trend reaching 50.7 μg/mL for the water group and 25.8 μg/mL the FO group, respectively.

**Figure 4:**
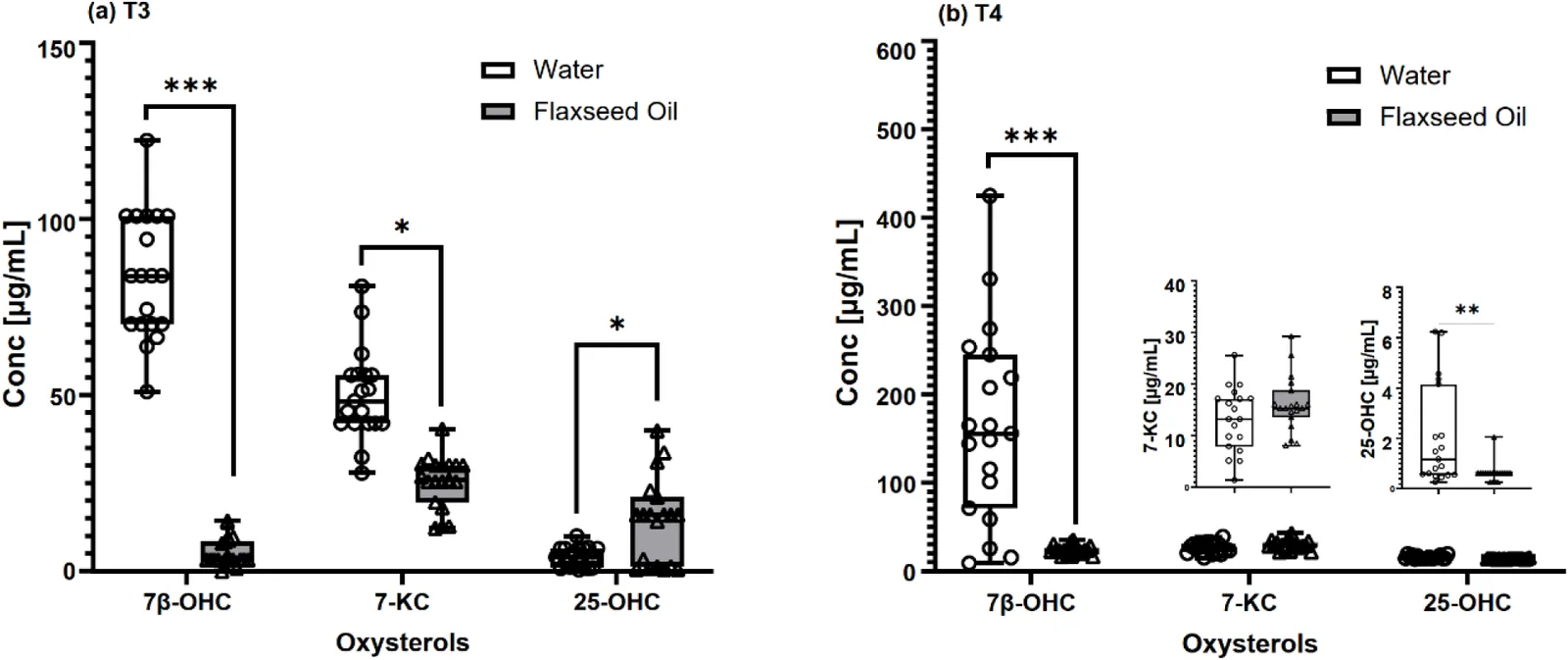
Comparison of 7β-OHC, 7-KC and 25-OHC [μg/mL] in participants who consumed water vs FO at time points T3 (a) and T4 (b). Data are presented as mean ± SD (*n* = 19); asterisk (*) indicates significant difference *p* < 0.05 between both groups determined by using paired multiple *t* test and Holm-Sidak’s multiple comparison test.

Immediately postexercise (T3) the water group exhibited a classic “oxidative burst” pattern: median plasma 7β–OHC climbed above 84 μg mL^–1^, with several individuals exceeding 120 μg mL^–1^, whereas the FO group remained near baseline (5.4 μg mL^–1^). The same, albeit attenuated, trend is seen for 7-KC and 25-OHC. Because the box–whisker distributions are largely nonoverlapping, the *t*-tests yield very large effect sizes (*p* < 0.001 for 7β-OHC; *p* < 0.05 for 7-KC and 25-OHC). From a data-perspective, these plots reflect a strong treatment × time interaction. The interquartile range (IQR) in the water group is roughly an order of magnitude wider for 7β-OHC than for the FO group, yet individual leverage points remain inside the 1.5 IQR whiskers, indicating that the difference is biological rather than artifactual. Such heteroscedasticity would inflate Type-I error in an unpaired design, but the present crossover structure and Holm–Šidák correction (α = 0.05) ensure robust significance despite multiple testing.

However, 15 min postexercise, a steep decline in 7-KC and 25-OHC occurred in the FO group, while 7β-OHC remained elevated in controls (Figure 4b). 7β-OHC in the plasma of individuals who received water rose even further compared to post exercise. It centered around 200 μg mL^–1^ with several extreme values >400 μg mL^–1^, whereas the level in the FO group remained suppressed (*p* < 0.001). By contrast, 7-KC partially converged for both groups, this might suggest multiple physical, metabolic, or enzymatic effects. Plasma muscular of subjects who have done HIIT was reported to decrease by approximately 16% post exercise to remain approximately 6% below baseline after 30 min of rest.^52^ This condition might have artificially influenced plasma oxysterol measurements at T4, independent of antioxidant status. And its concentrations fell in both groups during recovery, and the difference of 7-KC between the water and FO groups became nonsignificant at T4. The boxplot “re-compression” thus visualizes metabolic clearance: nonenzymatic species (7β-OHC, 7-KC) were cleared or transformed within minutes, while 25-OHC reacted differently.

Among the three oxysterols measured, 25-OHC showed a distinctly different pattern from 7β–OHC and 7-KC at T3, both in direction and in the biological process it reflects. In the FO group, concentrations were significantly elevated immediately post-HIIT (T3) but declined sharply during the 15 min recovery period (T4). The water group showed the reverse pattern, lower 25-OHC at T3, yet maintained relatively stable, modestly higher concentrations at T4 compared to the FO group, resulting in a statistically significant reversal of the between-group difference (T3: FOT > WAT, *p* < 0.05; T4: WAT > FOT, *p* < 0.01). This crossover pattern indicates that 25-OHC production in the FO group was tightly coupled to the exercise stimulus, whereas the water group showed a persistent, low-level 25-OHC signal extending into the recovery period.

Overall, the profound suppression of oxysterols in the FOT group indicates that FO supplementation provides protection against exercise-induced cholesterol oxidation. This temporal pattern indicates that antioxidant-rich FO intervention not only reduced the peak ROS burden but might accelerate resolution and clearance of oxidation products. The LC-MS/MS analysis was unable to detect the presence of the three oxysterols at time point T2 in both the group that consumed FO and the water. First, this is presumably due to insufficient sensitivity of the method used, which was optimized for the analysis of plasma with a high level of oxysterols. Second, it might be caused by the nature of oxysterol metabolism itself which is complex and shows multiple pathways of formation. Arguably, this discrepancy may be due to the fact that in this study, oxysterols were measured immediately after intense exercise in healthy human plasma, whereas in other publications, oxysterols are often analyzed in patients with chronic conditions such as Niemann-Pick disease,^53^ atherosclerosis, and coronary artery disease.^54^

### 3.3 Untargeted Phospholipidomics: Broader Remodeling of the Plasma Lipidome

Untargeted lipidomics allow for a comprehensive assessment of plasma lipid remodeling. Here, OPLS-DA models of the phospholipidome illustrated clear group separation at T2 and T3 (Figure 5a,b). Overall, our method found 130 phospholipids from diverse groups (PC, PE, PG, PI) corresponding to compounds at level 2 confidence according to Metabolomics Standard Initiative.^40^ Roughly two-thirds of significant PC species were elevated in the FO group, consistent with reduced levels of lipid degradation and oxidative damage. The heatmap analysis at T2 and T3 reinforced these trends, showing that most PCs had higher intensities in the FO group (Figure S4 and S5). However, at T4, the group separation vanished, mirroring the oxPC and oxysterol trends. These convergences suggest that plasma lipid fluctuation caused by high-performance exercise is largely acute and reversible but modulated in magnitude by antioxidant intake.

**Figure 5:**
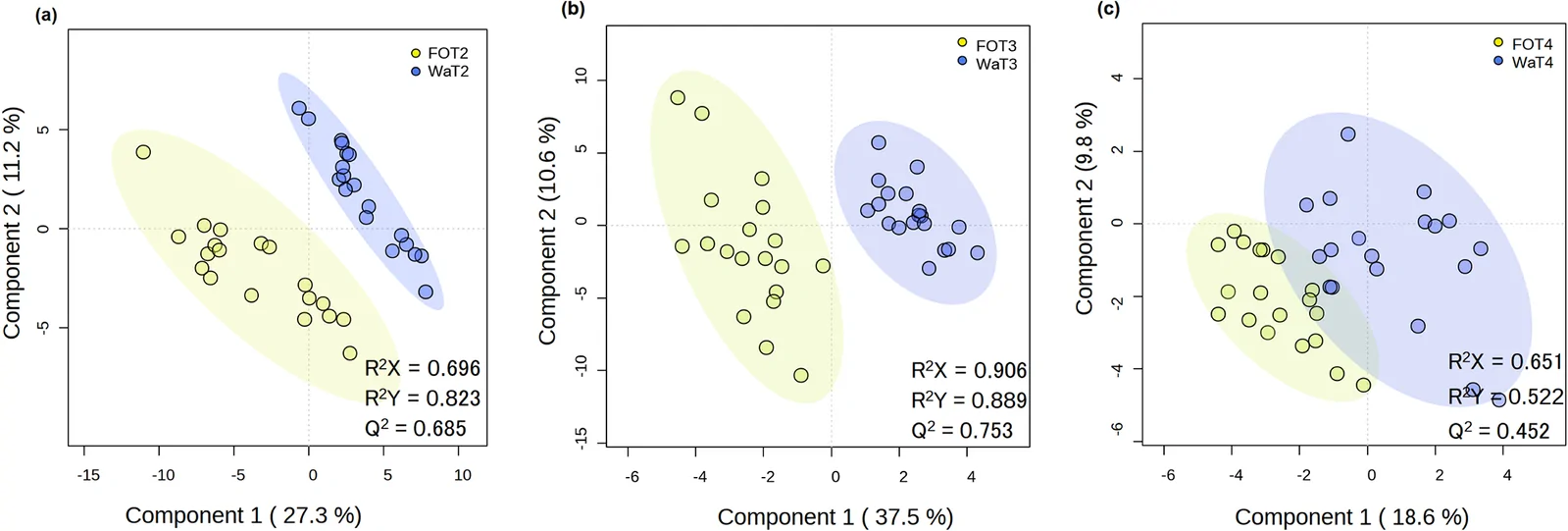
Multivariate analysis (OPLS-DA) using the data set of phospholipidome as features for comparison between (a) ΔT2 (T2–T1), (b) ΔT3 (T3–T1), and (c) ΔT4 (T4–T1) with the baseline of T1 for the individuals who consumed water (WaT) vs FO (FOT).

Notably, several oxPLs carrying oxylipin-like fatty acyl chains species were identified in the untargeted mode, including PG (18:1_15-HETE), PC (14:0_20-HDoHE), and PC (18:1_9-HODE) as shown in Figure S6. These lipids have known signaling functions, 15-HETE is described as anti-inflammatory,^55^ HODE is linked to oxidative injury,^56^ and their differential expression in treatment groups highlights the added value of broad lipidomic profiling. While our current study did not absolutely quantify oxylipins, a previous study found 9/13/15-HODE regioisomers hydroxy products as the dominating oxylipins in FO, reaching approximately up to 2.7 μmol/g.^57^ Furthermore, the untargeted lipidomic fingerprint revealed that plasma levels of several PC species were differentially modulated between the treatment and control groups, indicating a broader remodeling of membrane lipid composition.

These results provide compelling evidence that dietary antioxidant-rich FO supplementation can acutely modulate oxidative-stress biomarkers and lipid metabolism in response to exercise-induced ROS. They provide new mechanistic insights into how polyphenol-rich FO may confer systemic protection through the suppression of lipid peroxidation in plasma. Moreover, this study advances oxidative stress theory by demonstrating that targeted and untargeted lipidomics can detect rapid, diet-modulated shifts in oxidative lipid species in a controlled physiological stress model.

### 3.4 Phospholipidomics and Its Correlation to OxPCs and Oxysterols

Pairwise plot of the phospholipidome against oxPC is illustrated in Figure 6A, indicating the bulk of the intact PC species (red) cluster on the positive side of component 1, whereas all quantified oxPCs (blue) lie on the negative side. The angular separation of ∼180° translates into strong negative correlations (Spearman r ≈ −0.70 to −0.88). Subjects whose plasma contained higher levels of polyunsaturated PCs such as PC 38:4, PC 36:4, and PC-O 38:5 invariably exhibited lower concentrations of truncated aldehydic products KOOA-PC, KDdiA-PC and SONPC. The analyses presented in Figure 6 describe the covariation structure and are not the basis of the causal claim. Causal attribution in this study rests on the design. This mirror-image pattern mechanistically supports our earlier observation that polyphenol-rich FO preserved membrane PCs while simultaneously preventing their oxidative cleavage. A similar antagonistic topology can be observed in the pairwise plot of phospholipidome against oxysterols (Figure 6C), the nonenzymatic sterol oxidation markers 7-ketocholesterol (KC7_T3) and 7β-hydroxycholesterol (OHC7β_T3) project opposite to most intact PCs. Hence, protection of the PC pool appears to coincide with protection of cholesterol from free-radical attack, consistent with the shared lipid-peroxidation chain mechanism.

**Figure 6:**
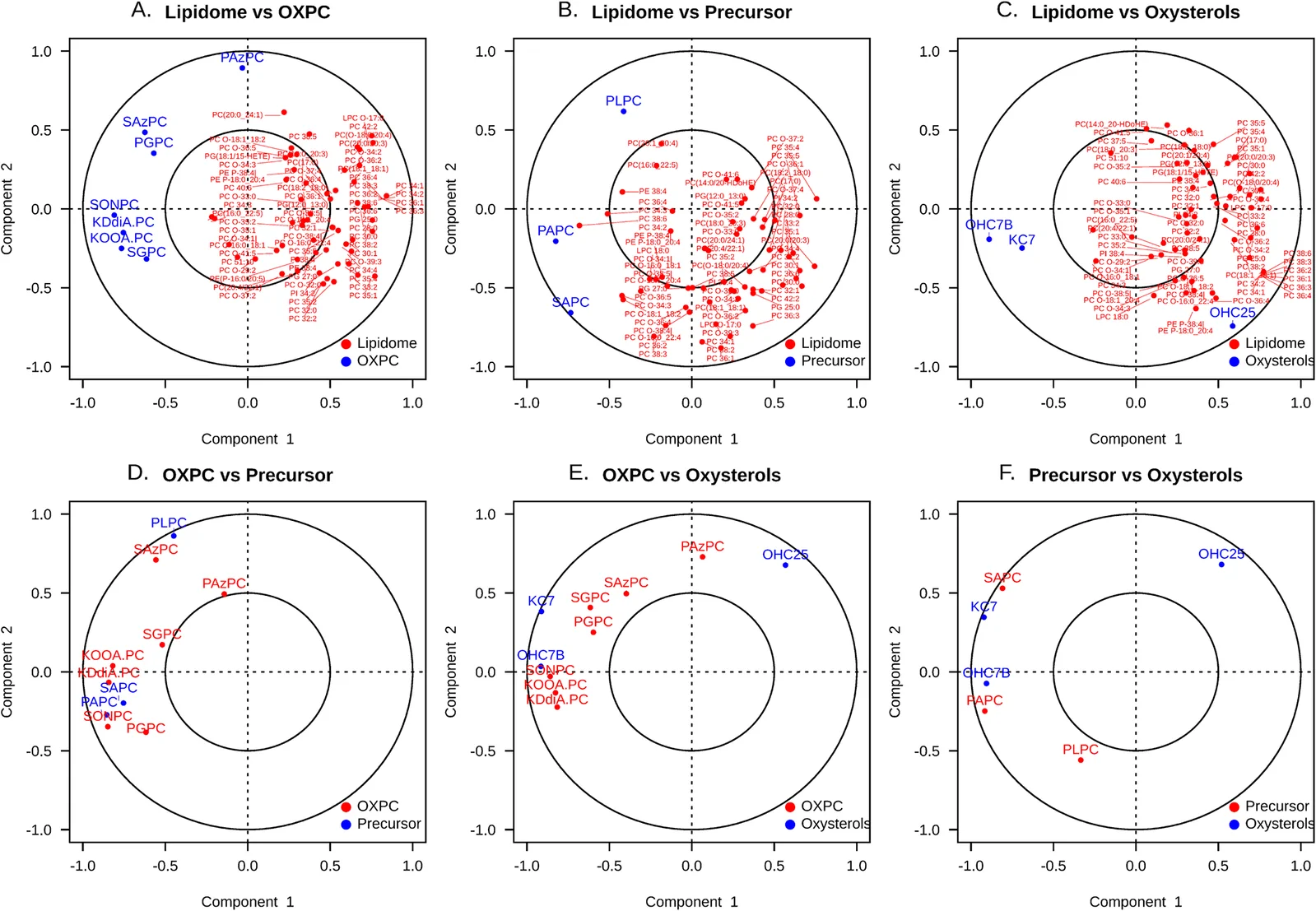
Pairwise Sparse Partial Least Squares (PLS) correlation plots illustrating cross-domain relationships among lipid subclasses in plasma at postexercise (T3) time point. Each panel depicts associations between two data sets: (A) lipidome vs oxPCs, (B) lipidome vs precursor PCs, (C) lipidome vs oxysterols, (D) oxPCs vs precursor PCs, (E) oxPCs vs oxysterols, and (F) precursor PCs vs oxysterols. The positions of variables represent their loadings on the first two latent components.

The lipidome versus precursor plot (Figure 6B) presents a more structurally nuanced picture. PAPC and SAPC, both containing arachidonic acid (20:4 n-6), project into the lower left quadrant, while PLPC containing linoleic acid (18:2 n-6) projected prominently upward into the upper left region. This divergence between the arachidonic acid-containing species and the linoleic acid-containing species may have biological significance. It appears that PLPC, PAPC, and SAPC do not covary identically with the broader lipidome, suggesting that their individual relationships with other plasma lipid species may differ and that the mechanisms underlying their respective changes in the FOT group might not be equivalent, which might answer the anomaly in PC values at T3 Figure 2b.

Furthermore, the pairwise oxPC against the PC precursor (Figure 6D) elucidates the immediate chemical lineage between intact substrates and their oxidized derivatives. This lineage may describe the chemical relationship between substrate and product and is not evidence of protection, which instead follows from the dissociation between precursor and product levels at ΔT2 presented in Section 3.1. All these PCs were located in the left quadrant in the point of view of component 1. Both data sets project exclusively into the left half of the correlation circle, suggesting a broadly positive covariation between the nonoxidized precursor PCs and their oxidized counterparts across study subjects. Conversely, precursor vs oxysterols (Figure 6F) revealed a near-orthogonal arrangement (≈ 90°), suggesting that while precursor PCs were prone to oxidation and oxysterols were highly unstable due to ROS exposure. Their quantitative relationships might be indirect and could be modulated by distinct enzymatic repair routes (e.g., cholesterol 25-hydroxylase for 25-OHC).

The oxPC vs Oxysterol (Figure 6E) plot supports the concept of a concurent oxidative response at peak physiological stress: KDdiA-PC and KOOA-PC project near KC7 with a small intervector angle (≤30°), consistent with a positive cross-subject association (r ≈ +0.6) and suggesting that truncated oxPC formation and 7-ketocholesterol accumulation are correlating under the HIIT condition. This is biologically plausible as lipid peroxidation is sustained by reactive lipid peroxyl (LOO•) and alkoxyl (LO•) radicals which can propagate chain reactions within lipoprotein lipid compartments, oxidizing both polyunsaturated phospholipids and cholesterol and thereby generating phospholipid- and sterol-derived oxidation products within the same oxidative episode. From a nutritional biochemistry perspective, the concerted attenuation of oxPCs and oxysterols observed in the polyphenol-enriched FO arm is consistent with reduced propagation of lipid peroxidation during acute oxidative challenge, rather than requiring direct attribution to a specific lipoprotein subclass or apolipoprotein microdomain.^58^ As discussed by Ghzaiel et al.,^59^ exercise and metabolic stress can rapidly trigger lipid peroxidation cascades within plasma and lipoprotein fractions. Therefore, our results advance the understanding of how targeted dietary strategies can acutely remodel the plasma “oxi-lipidome” and may inform precision nutrition approaches for athletes and at-risk populations.

Figure 7 depicts every cross-block association whose absolute Spearman coefficient exceeded 0.50 at T3. Ribbon hue encodes the sign of the correlation (red = positive, blue = negative), while the two outer polylines trace the relative trends of the mean component 1/2 scores for the FO group (FOT, blue) and the water control (WAT, orange). Negative correlation between PC and the other oxidation products dominates the network. More than two-thirds of the blue ribbons originate from polyunsaturated PCs in the lipidome sector and connect either the oxPC (e.g., KDdiA-PC, SGPC) or oxysterol (e.g., KC7, OHC25) sectors. This global anticorrelation mirrors the diametrically opposed vectors in the Lipidome vs oxPC and Lipidome vs Oxysterol PLS plots (Figure 6A, 6C), confirming that preservation of intact PCs is systematically accompanied by lower burdens of both phospholipid- and cholesterol-derived oxidation products.

**Figure 7:**
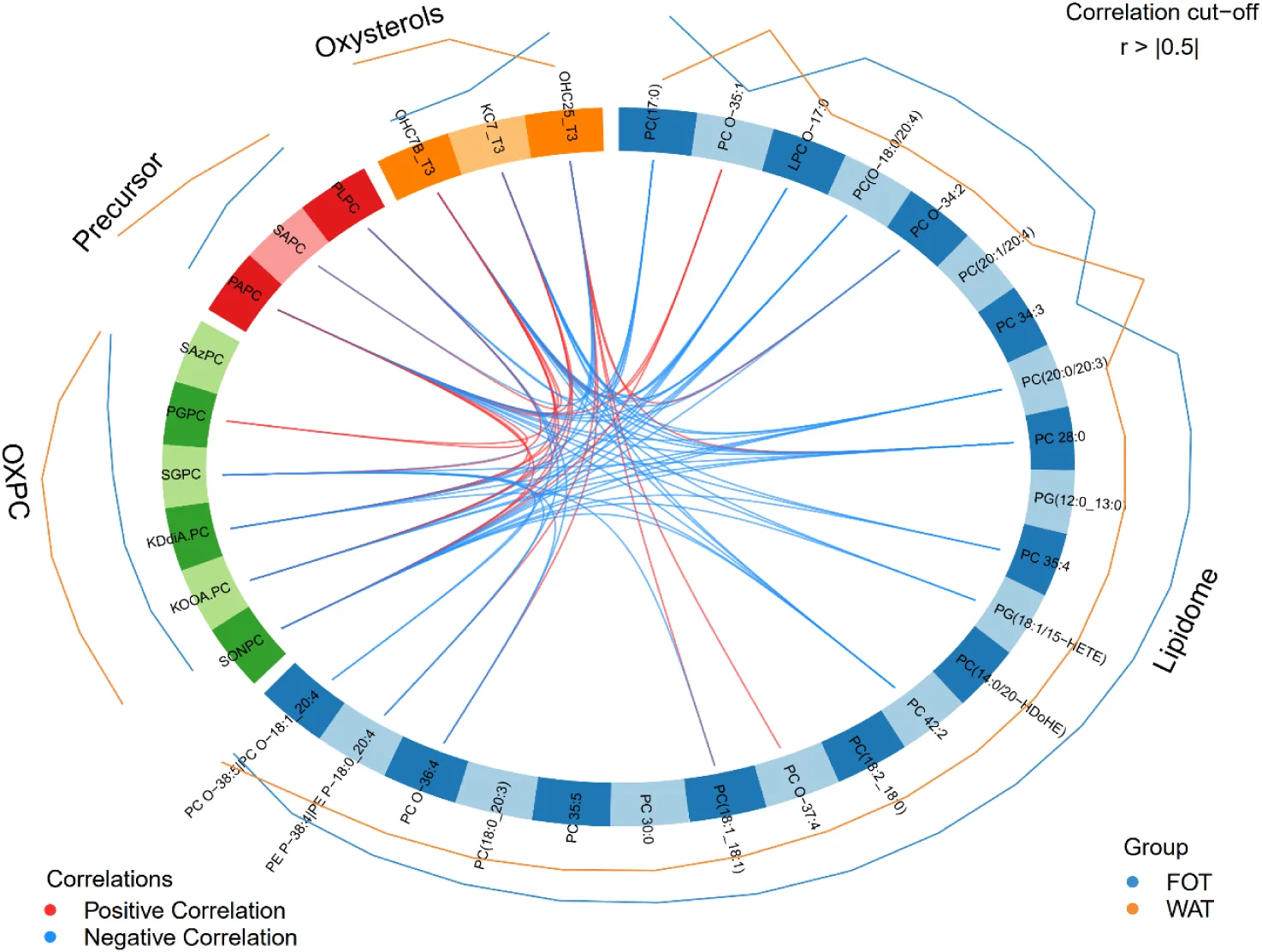
Circos plot illustrating cross-domain relationships between lipid subclasses in plasma at the postexercise (T3) time point. It illustrates correlations between oxPC precursors, oxysterols, and lipidome components. The plot displays positive (red) and negative (blue) correlations (r > |0.5|). Individual sectors represent different lipid species.

Positive substrate–product ribbons are possibly confined to direct chemical lineages. The tight red bundle connecting PAPC, PLPC and SAPC (precursor sector) with their fragmentation homologues PAzPC and SAzPC (oxPC sector) confirms the positive loadings in Figure 6D. The limited reach of these red ribbons indicates that, beyond the immediate cleavage pairings, intact PCs do not positively correlate with downstream oxysterols, consistent with the orthogonal geometry observed in Figure 6F. A radial hub links the most reactive oxPCs to oxysterols. Short, sturdy red ribbons join KDdiA-PC/KOOA-PC to KC7, highlighting synchronized generation of truncated aldehydes and 7-KC, a visual recapitulation of the oxPC vs Oxysterol convergence seen in Figure 6E. Together, Figures 6 and 7 provide complementary, multiscale evidence that the polyphenol-enriched FO intervention suppressed the oxidation induced by HIIT, preserving membrane integrity in human plasma samples. However, it should be noted that the analyses presented in Figures 6 and 7 describe covariation structure and are not the basis of the causal claim. Causal attribution in this study rests on the design.

### 3.5 Implications and Future Directions

This study provides a comprehensive lipid-based perspective on the mode of action of antioxidant-enriched FOs modulating lipid oxidation dynamics under acute physiological stress. This controlled, crossover trial is the first to interrogate *in vivo* redox kinetics across two lipid classes, i.e., oxPCs and oxysterols, complemented by untargeted lipidomic profiling of the broader plasma lipidome by integrating targeted LC-MS/MS quantification with chemometrics-guided multivariate modeling. It supports and extends existing oxidative stress theory by demonstrating lipid-class selectivity of antioxidant protection (e.g., oxPCs vs PCs vs oxysterols). And expressing immediate metabolic remodeling of plasma lipids following dietary antioxidant-rich FO intervention. The results imply that preexercise nutritional strategies can substantially modify the oxidative landscape of the human body. These findings lead the way for mechanistic studies linking lipid oxidation and an antioxidative diet and may inform dietary recommendations for athletes or individuals at cardiovascular risk.

Longer-term supplementation with flaxseed oil and with α-linolenic acid has been examined for other classes of oxidation product, with flaxseed oil increasing plasma F1-phytoprostanes in healthy men,^28^ and α-linolenic acid-rich supplements altering plasma oxylipin profiles in a time-dependent and sex-dependent manner,^29,30^ Those products and those time scales differ from the oxPCs and oxysterols measured here, and the relationship between the two is not established. Linking them would require a repeated-dose design in which the acute response is measured at intervals across a supplementation period, so that a shift in the acute response with continued intake can be separated from a change in steady-state concentrations.

Future studies should extend recovery time points, hematocrit, hemoglobin, integrate transcriptomic and proteomic layers, and explore sex-specific and dose–response effects. Therefore, absolute plasma concentrations can be corrected for exercise-induced volume shifts. Under the acute oxidative pressure of high-intensity interval exercise, the phenolic-enriched FO matrix almost completely prevented the surge of the kinetically sensitive markers, 7β-hydroxy-cholesterol and electrophilic oxPCs, additionally attenuating the rise of 7-ketocholesterol, but not the same case for 25-hydroxycholesterol. OPLS-DA score plots corroborated these biochemical findings by resolving clear treatment-dependent clustering as early as 2 h postingestion and amplifying the separation at peak oxidative stress. Together, the quantitative and multivariate data demonstrate that the intervention acts at the initiation phase of lipid oxidation rather than merely accelerating product clearance. Untargeted lipidomics expanded these observations, revealing differential modulation of phospholipidome, including oxidized molecular species carrying oxylipin-like acyl chains. These lipids are known to participate in redox signaling and inflammatory resolution pathways. Together, the targeted and untargeted data sets converge to demonstrate that the antioxidant-enriched FO acts effectively stabilizing redox-sensitive lipid pools and preventing the propagation of secondary oxidative products. The findings provide novel *in vivo* evidence that functional lipid matrices rich in polyphenols can serve as effective nutritional strategies to mitigate oxidative damage in humans whether triggered by exercise or occurring independently of it.

## Supplementary Material



## Data Availability

The data that support the findings of this study are available from the corresponding author upon reasonable request.

## Notes

Supporting Information 1: the list of MRM transitions of oxidized and PC compounds, SPLASH Lipidomix Internal Standards, oxysterols (25-OHC, 7β-OHC, and 7KC), example QC curve for the lipid feature used for data normalization and processing, and VIP scores from the OPLS-DA model as the dominant discriminatory features between different time points, as well as the heatmap showing the normalized peak intensity levels of individual lipid species across WaT and FOT from untargeted lipidomics at T2, and, boxplots showing the normalized intensity of selected lipid metabolites across FOT and WAT groups at four time points; Supporting Information 2 and 3: the lipidomics standard reporting checklists according to the International Lipidomics Society (PDF)

## List of Abbreviations

HDoHE: hydroxydocosahexaenoic acid
HETE: hydroxyeicosatetraenoic acid
HODE: 1-palmitoyl-2-(5-hydroxy-8-oxo-6-octenoyl)-*sn*-glycero-3-phosphatidylcholine
HOOA-PC: 1-palmitoyl-2-(5-hydroxy-8-oxo-6-octenoyl)-*sn*-glycero-3-phosphatidylcholine
KDdiA-PC: 1-palmitoyl-2­(4′-keto-dodec-3′-ene-dioyl)-*sn*-glycero-3-phosphocholine
KOdiA-PC: 1-palmitoyl-2-(5′-keto-6′-octenedioyl)-*sn*-glycero-3-phosphocholine
KOOA-PC: 1-palmitoyl-2-(5-hydroxy-8-oxo-6-octenoic acid)-*sn*-glycero-3-phospatidylocholine
MRM: multiple reaction monitoring
PAPC: 1-palmitoyl-2-arachidonoyl-*sn*-glycero-3-phosphatidylcholine
PAzPC: 1-palmitoyl-2-azelaoyl-*sn*-glycero-3-phosphatidylcholine
PGPC: 1-palmitoyl-2-glutaroyl-*sn*-glycero-3-phosphatidylcholin
PL: Phospholipid
PLPC: 1-palmitoyl-2-linoleoyl-*sn*-glycero-3-phosphatidylcholine
PONPC: 1-palmitoyl-2-(9′-oxo-nonanoyl)-*sn*-glycero-3-phosphatidylcholine
POVPC: 1-palmitoyl-2-(5′-oxo-valeroyl)-*sn*-glycero-3-phosphatidylcholine
SAPC: 1-stearoyl-2-arachidonoyl-*sn*-glycero-3-phosphatidylcholine
SAzPC: 1-stearoyl-2-azelaoyl-*sn*-glycero-3-phosphatidylcholine
SGPC: 1-stearoyl-2-glutaroyl-*sn*-glycero-3-phosphatidylcholine
SLPC: 1-stearoyl-2-linoleoyl-*sn*-glycero-3-phosphatidylcholine
SONPC: 1-stearoyl-2-(9′-oxo-nonanoyl)-*sn*-glycero-3-phosphatidylcholine

## References

[1] Parikh M., Maddaford T. G., Austria J. A., Aliani M., Netticadan T., Pierce G. N. (2019). Dietary Flaxseed as a Strategy for Improving Human
Health. Nutrients.

[2] Rodriguez-Leyva D., Bassett C. M. C., McCullough R., Pierce G. N. (2010). The Cardiovascular
Effects of Flaxseed and Its Omega-3 Fatty Acid, Alpha-Linolenic Acid. Can. J. Cardiol..

[3] Kajla P., Sharma A., Sood D. R. (2015). Flaxseed—a
Potential Functional
Food Source. J. Food Sci. Technol..

[4] Ma Y., Liu X., Sun H., Wang Y., Bai G., Guo Q., Xiao S., Peng Y., Song L., Qiao M., Huang X. (2025). Preparation
of Flaxseed Oil Nanoemulsion and Its Effect on Oxidation
Stability of Flaxseed Oil and Prediction of Shelf Life. LWT.

[5] Fruehwirth S., Steinschaden R., Woschitz L., Richter P., Schreiner M., Hoffmann B., Hoffmann W., Pignitter M. (2020). Oil-Assisted
Extraction of Polyphenols from Press Cake to Enhance Oxidative Stability
of Flaxseed Oil. LWT.

[6] Zhang Z., Guo J. (2025). Deciphering Oxidative Stress in Cardiovascular
Disease Progression:
A Blueprint for Mechanistic Understanding and Therapeutic Innovation. Antioxidants.

[7] Chen X., Xie N., Feng L., Huang Y., Wu Y., Zhu H., Tang J., Zhang Y. (2025). Oxidative Stress in Diabetes Mellitus
and Its Complications: From Pathophysiology to Therapeutic Strategies. Chin Med. J. Engl..

[8] Scarian E., Viola C., Dragoni F., Di Gerlando R., Rizzo B., Diamanti L., Gagliardi S., Bordoni M., Pansarasa O. (2024). New Insights into Oxidative Stress
and Inflammatory Response in Neurodegenerative Diseases. Int. J. Mol. Sci..

[9] Poznyak A. V., Nikiforov N. G., Markin A. M., Kashirskikh D. A., Myasoedova V. A., Gerasimova E. V., Orekhov A. N. (2021). Overview of OxLDL
and Its Impact on Cardiovascular Health: Focus on Atherosclerosis. Front. Pharmacol..

[10] Munno M., Mallia A., Greco A., Modafferi G., Banfi C., Eligini S. (2024). Radical Oxygen Species, Oxidized
Low-Density Lipoproteins, and Lectin-like Oxidized Low-Density Lipoprotein
Receptor 1: A Vicious Circle in Atherosclerotic Process. Antioxidants.

[11] Jiang H., Zhou Y., Nabavi S. M., Sahebkar A., Little P. J., Xu S., Weng J., Ge J. (2022). Mechanisms of Oxidized LDL-Mediated
Endothelial Dysfunction and Its Consequences for the Development of
Atherosclerosis. Front Cardiovasc Med..

[12] Bochkov V. N., Oskolkova O. V., Birukov K. G., Levonen A.-L., Binder C. J., Stöckl J. (2010). Generation and Biological Activities of Oxidized Phospholipids. Antioxid. Redox Signal..

[13] Levitan I., Volkov S., Subbaiah P. V. (2010). Comprehensive
Invited Review Oxidized
LDL: Diversity, Patterns of Recognition, and Pathophysiology. Antioxid. Redox Signal..

[14] Santa D. E., Brown T. P., Im W., Wittenberg N. J. (2024). Atherosclerotic
Oxidized Lipids Affect Formation and Biophysical Properties of Supported
Lipid Bilayers and Simulated Membranes. J. Phys.
Chem. B.

[15] Solati Z., Surendran A., Edel A., Roznik M., Allen D., Ravandi A. (2021). Increase in Plasma Oxidized Phosphatidylcholines
(OxPCs)
in Patients Presenting With ST-Elevation Myocardial Infarction (STEMI). Front. Med..

[16] Karki P., Birukov K. G. (2020). Oxidized Phospholipids
in Healthy and Diseased Lung
Endothelium. Cells.

[17] Yutuc E., Dickson A. L., Pacciarini M., Griffiths L., Baker P. R. S., Connell L., Öhman A., Forsgren L., Trupp M., Vilarinho S., Khalil Y., Clayton P. T., Sari S., Dalgic B., Höflinger P., Schöls L., Griffiths W. J., Wang Y. (2021). Deep Mining of Oxysterols and Cholestenoic Acids in Human Plasma
and Cerebrospinal Fluid: Quantification Using Isotope Dilution Mass
Spectrometry. Anal. Chim. Acta.

[18] Anderson A., Campo A., Fulton E., Corwin A., Jerome W. G., O’connor M. S. (2020). 7-Ketocholesterol
in Disease and Aging. Redox Biol..

[19] Luquain-Costaz C., Delton I. (2024). Oxysterols in Vascular
Cells and Role in Atherosclerosis. Adv. Exp.
Med. Biol..

[20] Moslemi E., Dehghan P., Khani M., Sarbakhsh P., Sarmadi B. (2023). The Effects of Date Seed (Phoenix
Dactylifera) Supplementation
on Exercise-Induced Oxidative Stress and Aerobic and Anaerobic Performance
Following High-Intensity Interval Training Sessions: A Randomised,
Double-Blind, Placebo-Controlled Trial. Br.
J. Nutr..

[21] Kruk J., Aboul-Enein B. H., Duchnik E., Marchlewicz M. (2022). Antioxidative
Properties of Phenolic Compounds and Their Effect on Oxidative Stress
Induced by Severe Physical Exercise. J. Physiol.
Sci..

[22] McAnulty S. R., McAnulty L. S., Nieman D. C., Dumke C. L., Morrow J. D., Utter A. C., Henson D. A., Proulx W. R., George G. L. (2004). Consumption
of Blueberry Polyphenols Reduces Exercise-Induced Oxidative Stress
Compared to Vitamin C. Nutr. Res..

[23] Taub P. R., Ramirez-Sanchez I., Patel M., Higginbotham E., Moreno-Ulloa A., Román-Pintos L.
M., Phillips P., Perkins G., Ceballos G., Villarreal F. (2016). Beneficial
Effects of Dark Chocolate on Exercise Capacity in Sedentary Subjects:
Underlying Mechanisms. A Double Blind, Randomized, Placebo Controlled
Trial. Food Funct..

[24] Tyurina Y. Y., Tyurin V. A., Anthonymuthu T., Amoscato A. A., Sparvero L. J., Nesterova A. M., Baynard M. L., Sun W., He R. R., Khaitovich P., Vladimirov Y. A., Gabrilovich D. I., Bayır H., Kagan V. E. (2019). Redox Lipidomics Technology: Looking
for a Needle in a Haystack. Chem. Phys. Lipids.

[25] Fruehwirth S., Zehentner S., Salim M., Sterneder S., Tiroch J., Lieder B., Zehl M., Somoza V., Pignitter M. (2020). In Vitro Digestion of Grape Seed Oil Inhibits Phospholipid-Regulating
Effects of Oxidized Lipids. Biomolecules.

[26] Barker-Tejeda T. C., Villaseñor A., Gonzalez-Riano C., López-López Á., Gradillas A., Barbas C. (2021). In Vitro Generation of Oxidized Standards
for Lipidomics. Application to Major Membrane Lipid Components. J. Chromatogr. A.

[27] Hornburg D., Wu S., Moqri M., Zhou X., Contrepois K., Bararpour N., Traber G. M., Su B., Metwally A. A., Avina M., Zhou W., Ubellacker J. M., Mishra T., Schüssler-Fiorenza
Rose S. M., Kavathas P. B., Williams K. J., Snyder M. P. (2023). Dynamic
Lipidome
Alterations Associated with Human Health, Disease and Ageing. Nat. Metab..

[28] Barden A. E., Croft K. D., Durand T., Guy A., Mueller M. J., Mori T. A. (2009). Flaxseed Oil Supplementation Increases
Plasma F1-Phytoprostanes
in Healthy Men. J. Nutr..

[29] Gabbs M., Zahradka P., Taylor C. G., Aukema H. M. (2021). Time Course and
Sex Effects of α-Linolenic Acid-Rich and DHA-Rich Supplements
on Human Plasma Oxylipins: A Randomized Double-Blind Crossover Trial. J. Nutr..

[30] Pauls S. D., Rodway L. R., Sidhu K. K., Winter T., Sidhu N., Aukema H. M., Zahradka P., Taylor C. G. (2021). Oils Rich in α-Linolenic
Acid or Docosahexaenoic Acid Have Distinct Effects on Plasma Oxylipin
and Adiponectin Concentrations and on Monocyte Bioenergetics in Women
with Obesity. J. Nutr..

[31] Gassner M., Bragagna L., Heidari H., Bayaz D., Stumpf-Knaus C., Schlosser L., Lemberg J., Brem J., Pignitter M., Strauss M. (2025). Acute Impact of Polyphenol-Rich vs. Carbohydrate-Rich
Foods and Beverages on Exercise-Induced ROS and FRAP in Healthy Sedentary
Female Adults — A Randomized Controlled Trial. Antioxidants.

[32] Ademowo O. S., Sharma P., Cockwell P., Reis A., Chapple I. L., Griffiths H. R., Dias I. H. K. (2020). Distribution of Plasma Oxidised Phosphatidylcholines
in Chronic Kidney Disease and Periodontitis as a Co-Morbidity. Free Rad. Biol. Med..

[33] Pellegrino R. M., Di Veroli A., Valeri A., Goracci L., Cruciani G. (2014). LC/MS Lipid
Profiling from Human Serum: A New Method for Global Lipid Extraction. Anal. Bioanal. Chem..

[34] Helmschrodt C., Becker S., Schröter J., Hecht M., Aust G., Thiery J., Ceglarek U. (2013). Fast LC–MS/MS
Analysis of
Free Oxysterols Derived from Reactive Oxygen Species in Human Plasma
and Carotid Plaque. Clin. Chim. Acta.

[35] Philippova M., Oskolkova O. V., Bicker W., Schoenenberger A. W., Resink T. J., Erne P., Bochkov V. N. (2019). Analysis of Fragmented
Oxidized Phosphatidylcholines in Human Plasma Using Mass Spectrometry:
Comparison with Immune Assays. Free Rad. Biol.
Med..

[36] Mcdonald J. G., Ejsing C. S., Kopczynski D., Holćapek M., Aoki J., Arita M., Arita M., Baker E. S., Bertrand-Michel J., Bowden J. A. (2022). Introducing the Lipidomics
Minimal Reporting Checklist. Nat. Metab..

[37] Cajka T., DeFelice B., Wohlgemuth G., Shen T., Fiehn O. (2023). Long-Term
Robustness of Agilent LC/Q-TOF Systems for Untargeted Lipidomics In
Large Plasma Cohort Studies for Clinical Research. Agil. Appl. Note Metabolomics Clin. Res..

[38] Aurum F. S., Imaizumi T., Thammawong M., Suhandy D., Praseptiangga D., Tsuta M., Nagata M., Nakano K. (2022). Lipidomic Profiling
of Indonesian Coffee to Determine Its Geographical Origin by LC–MS/MS. Eur. Food Res. Technol..

[39] Tsugawa H., Ikeda K., Takahashi M., Satoh A., Mori Y., Uchino H., Okahashi N., Yamada Y., Tada I., Bonini P., Higashi Y., Okazaki Y., Zhou Z., Zhu Z.-J., Koelmel J., Cajka T., Fiehn O., Saito K., Arita M., Arita M. (2020). A Lipidome Atlas in
MS-DIAL 4. Nat. Biotechnol..

[40] Fiehn O., Robertson D., Griffin J., van der Werf M., Nikolau B., Morrison N., Sumner L. W., Goodacre R., Hardy N. W., Taylor C., Fostel J., Kristal B., Kaddurah-Daouk R., Mendes P., van Ommen B., Lindon J. C., Sansone S.-A. (2007). The Metabolomics
Standards Initiative
(MSI). Metabolomics.

[41] Pang Z., Chong J., Li S., Xia J. (2020). MetaboAnalystR 3.0:
Toward an Optimized Workflow for Global Metabolomics. Metabolites.

[42] Broadhurst D., Goodacre R., Reinke S. N., Kuligowski J., Wilson I. D., Lewis M. R., Dunn W. B. (2018). Guidelines and Considerations
for the Use of System Suitability and Quality Control Samples in Mass
Spectrometry Assays Applied in Untargeted Clinical Metabolomic Studies. Metabolomics.

[43] Karki P., Birukov K. G. (2021). Oxidized Phospholipids in Control
of Endothelial Barrier
Function: Mechanisms and Implication in Lung Injury. Front. Endocrinol..

[44] Bochkov V., Gesslbauer B., Mauerhofer C., Philippova M., Erne P., Oskolkova O. V. (2017). Pleiotropic Effects of Oxidized Phospholipids. Free Rad. Biol. Med..

[45] Myburgh K. H. (2014). Polyphenol
Supplementation: Benefits for Exercise Performance or Oxidative Stress?. Sport. Med..

[46] Zhou Z., Chen C., Teo E. C., Zhang Y., Huang J., Xu Y., Gu Y. (2022). Intracellular
Oxidative Stress Induced by Physical
Exercise in Adults: Systematic Review and Meta-Analysis. Antioxidants.

[47] Huang F., Wang K., Shen J. (2020). Lipoprotein-Associated Phospholipase
A2: The Story Continues. Med. Res. Rev..

[48] Henning T., Kochlik B., Kusch P., Strauss M., Jurić V., Pignitter M., Marusch F., Grune T., Weber D. (2022). Pre-Operative
Assessment of Micronutrients, Amino Acids, Phospholipids and Oxidative
Stress in Bariatric Surgery Candidates. Antioxidants.

[49] Solati Z., Edel A. L., Shang Y., Karmin O., Ravandi A. (2018). Oxidized Phosphatidylcholines
Are Produced in Renal Ischemia Reperfusion Injury. PLoS One.

[50] Poli G., Leoni V., Biasi F., Canzoneri F., Risso D., Menta R. (2022). Oxysterols: From Redox
Bench to Industry. Redox Biol..

[51] Carre R., Vigne S., Pot C. (2025). 25-Hydroxycholesterol
in Inflammation. Curr. Opin. Endocr. Metab.
Res..

[52] Mandić M., Forsgren M. F., Romu T., Widholm P., Sundblad P., Gustafsson T., Rullman E. (2021). Interval-Induced Metabolic Perturbation
Determines Tissue Fluid Shifts into Skeletal Muscle. Physiol. Rep..

[53] Lin N., Zhang H., Qiu W., Ye J., Han L., Wang Y., Gu X. (2014). Determination of 7-Ketocholesterol
in Plasma by LC-MS for Rapid Diagnosis of Acid SMase-deficient Niemann-Pick
Disease. J. Lipid Res..

[54] Zmysłowski A., Szterk A. (2019). Oxysterols as a Biomarker
in Diseases. Clin. Chim. Acta.

[55] Kim B. J., Shin K. C., Oh D. K. (2014). Enzymatic
Production of 15-Hydroxyeicosatetraenoic
Acid from Arachidonic Acid by Using Soybean Lipoxygenase. J. Microbiol. Biotechnol..

[56] Evans W. A., Eccles-Miller J. A., Anderson E., Farrell H., Baldwin W. S. (2024). 9-HODE
and 9-HOTrE Alter Mitochondrial Metabolism, Increase Triglycerides,
and Perturb Fatty Acid Uptake and Synthesis Associated Gene Expression
in HepG2 Cells. Prostaglandins, Leukot. Essent.
Fat. Acids.

[57] Koch E., Wiebel M., Löwen A., Willenberg I., Schebb N. H. (2022). Characterization of the Oxylipin
Pattern and Other
Fatty Acid Oxidation Products in Freshly Pressed and Stored Plant
Oils. J. Agric. Food Chem..

[58] Dini I., Grumetto L. (2022). Recent Advances in
Natural Polyphenol Research. Molecules.

[59] Ghzaiel I., Sassi K., Zarrouk A., Ghosh S., Dias I. H. K., Nury T., Ksila M., Essadek S., Joutey M. T., Brahmi F. (2022). Sources
of 7-Ketocholesterol, Metabolism and Inactivation
Strategies: Food and Biomedical Applications. Redox Exp. Med..

[60] Dunn W. B., Broadhurst D., Begley P., Zelena E., Francis-McIntyre S., Anderson N., Brown M., Knowles J. D., Halsall A., Haselden J. N., Nicholls A. W., Wilson I. D., Kell D. B., Goodacre R. (2011). Procedures for Large-Scale Metabolic Profiling of Serum
and Plasma Using Gas Chromatography and Liquid Chromatography Coupled
to Mass Spectrometry. Nat. Protoc..

