# Supplementary material for: Oxidomics of Human Plasma Reveals Lower Oxidized Lipids After Acute Intake of Polyphenol-Enriched Flaxseed Oil: A Randomized Crossover Exercise Trial
Source: J Agric Food Chem. 2026 Sep 22;74(39):31240–52. doi: 10.1021/acs.jafc.6c08737 (PMC13651599; doi:10.1021/acs.jafc.6c08737)
Supplement: Supplementary file 1 [file jf6c08737_si_001.pdf]

Supporting information

Oxidomics of Human Plasma Reveals Lower Oxidized Lipids after Acute Intake of Polyphenol-Enriched Flaxseed Oil: A Randomized Crossover Exercise Trial

Fawzan Sigma Aurum[1,5], Julia Brem[1], Matthias Strauss[1,2], Markus Gassner[3,6], Daniel König[3,4,6], Marc Pignitter[1*]

[1]Institute of Physiological Chemistry, Faculty of Chemistry, University of Vienna, Josef-Holaubek-Platz 2, 1090 Vienna, Austria

[2]Vienna Doctoral School in Chemistry (DoSChem), Faculty of Chemistry, University of Vienna, Währinger Str. 42, 1090 Vienna, Austria

[3]Department of Nutrition, Faculty of Life Sciences, Section for Nutrition, Exercise and Health, University of Vienna, 1090 Vienna, Austria

[4]Centre for Sport Science and University Sports, Department of Sports Science, Section for Nutrition, Exercise and Health, University of Vienna, Auf der Schmelz 6, 1150 Vienna, Austria

[5]Research Center for Food Technology and Processing, National Research and Innovation Agency, 55861 Yogyakarta, Indonesia

[6]Vienna Doctoral School of Pharmaceutical, Nutritional and Sport Sciences, University of Vienna, Josef-Holaubek-Platz 1090 Vienna, Austria

Orcid ID: F.S.A. (https://orcid.org/0000-0001-8422-2118), D.K. (https://orcid.org/0000-0001-7002-1286), M.S. (https://orcid.org/0000-0003-0078-1612), M.G. (https://orcid.org/0009-0006-8021-4528)

*Corresponding author: marc.pignitter@univie.ac.at (M.P. https://orcid.org/0000-0002-5793-8572)

Supplementary Material 1

Table S1. MRM transition of Oxidized and PC compounds

Compound	Precursor (m/z)	Product (m/z)	CE (eV)	Polarity
PAPC*	782	184	30	ESI+
PLPC*	758	184	30	ESI+
SAPC*	810	184	30	ESI+
SLPC*	786	184	30	ESI+
HOOA-PC	649	184	36	ESI+
KDdiA-PC	720	184	38	ESI+
KOOA-PC	648	184	34	ESI+
PAzPC	666	184	30	ESI+
PGPC	610	184	30	ESI+
PONPC	650	184	32	ESI+
POVPC	594	184	20	ESI+
SAzPC	694	184	30	ESI+
SGPC	638	184	28	ESI+
SONPC	678	184	38	ESI+

* Precursor PCs

Table S2. MRM transitions of SPLASH Lipidomix IS

Compound	Precursor (m/z)	Product (m/z)	CE (eV)	Polarity
18.1_d7_LPC	529.40	184.10	25	ESI+
18.1_d7_LPE	487.40	346.20	25	ESI+
18.1_d7_chol_ester	675.70	369.40	25	ESI+
15.0_18.1_d7_DG	605.60	346.30	25	ESI+
15.0_18.1_d7_TG	829.40	570.50	30	ESI+
cholesterol_d7	411.40	376.40	40	ESI+
15.1_18.1_d7_PC	753.60	482.30	35	ESI+
15.0_18.1_d7_PE	709.50	288.30	-35	ESI−
15.0_18.1_d7_PG	740.60	288.30	-40	ESI−
15.0_18.1_d7_PI	828.60	288.30	-60	ESI−

Table S3. MRM Transitions of 25-OHC, 7β-OHC and 7-KC (quant & qual)

Compound	Precursor (m/z)	Product (m/z)	CE (eV)	Polarity
25-hydroxycholesterol (quant)	385.00	367.00	13	ESI+
25-hydroxycholesterol (qual)	401.00	69.00	50	ESI+
7β-hydroxycholesterol (quant)	401.34	69.00	50	ESI+
7β-hydroxycholesterol (qual)	385.35	83.00	40	ESI+
7-ketocholesterol (quant)	401.34	95.00	40	ESI+
7-ketocholesterol (qual)	385.00	367.00	13	ESI+

Table S4. MRM Transitions of 25-OHC-d6, 7β-OHC-d7 and 7-KC-d7 (quant & qual)

Compound	Precursor (m/z)	Product (m/z)	CE (eV)	Polarity
25-hydroxycholesterol-d6 (quant)	391.00	373.00	10	ESI+
25-hydroxycholesterol-d6 (qual)	391.00	161.00	33	ESI+
7β-hydroxycholesterol-d7 (quant)	374.60	159.10	20	ESI+
7β-hydroxycholesterol-d7 (qual)	374.30	147.40	22	ESI+
7-ketocholesterol-d7 (quant)	408.35	95.00	35	ESI+
7-ketocholesterol-d7 (qual)	408.35	81.00	32	ESI+

Table S5. Linear equations and R^2 values of oxysterol standards 7-KC, 7β-OHC and 25-OHC

Standard	linear equation	R^2
7-ketocholesterol	y = 0.68751 + 0.050719 * area	0.9910
7β-hydroxycholesterol	y = -4.62467 + 0.060590 * area	0.9286
25-hydroxycholesterol	y = 0.26390 + 0.003752 * area	0.9963

Table S6. Polyphenol content of the polyphenol-enriched flaxseed oil compared with conventional cold-pressed flaxseed oil from the same seed batch and press line.

**Parameter**	**Polyphenol-enriched FO**	**Conventional FO**	**Change**
Total polyphenols (mg GAE/100 g oil)	8.22 ± 0.08	0.68 ± 0.08	12-fold
**Individual polyphenols (relative abundance, AUC × 10^5)**			
Syringic aldehyde	2.31 ± 0.21	0.16 ± 0.02	+1384%
Chlorogenic acid	0.34 ± 0.01	0.10 ± 0.01	+233%
Ferulic acid	22.6 ± 1.92	9.78 ± 0.80	+131%
p-Coumaric acid	32.1 ± 2.43	16.4 ± 0.81	+95.6%
Secoisolariciresinol	0.47 ± 0.04	0.29 ± 0.01	+60.3%
Caffeic acid	6.16 ± 0.47	4.30 ± 0.20	+43.2%
Coniferyl aldehyde	4.04 ± 0.31	3.10 ± 0.23	+30.6%
Kaempferol	15.2 ± 0.71	14.0 ± 1.28	+8.25%
Catechin	4.20 ± 0.31	not detected	newly detectable
Vanillin	2.23 ± 0.20	1.95 ± 0.26	not significant
**Oxidative status**			
Peroxide value (meq O_2/kg oil)	1.79 ± 0.01	7.42 ± 0.27	−76%
Oxidation induction time (h)	1.39 ± 0.02	1.01 ± 0.02	+38%

Fruehwirth et al. (2020).

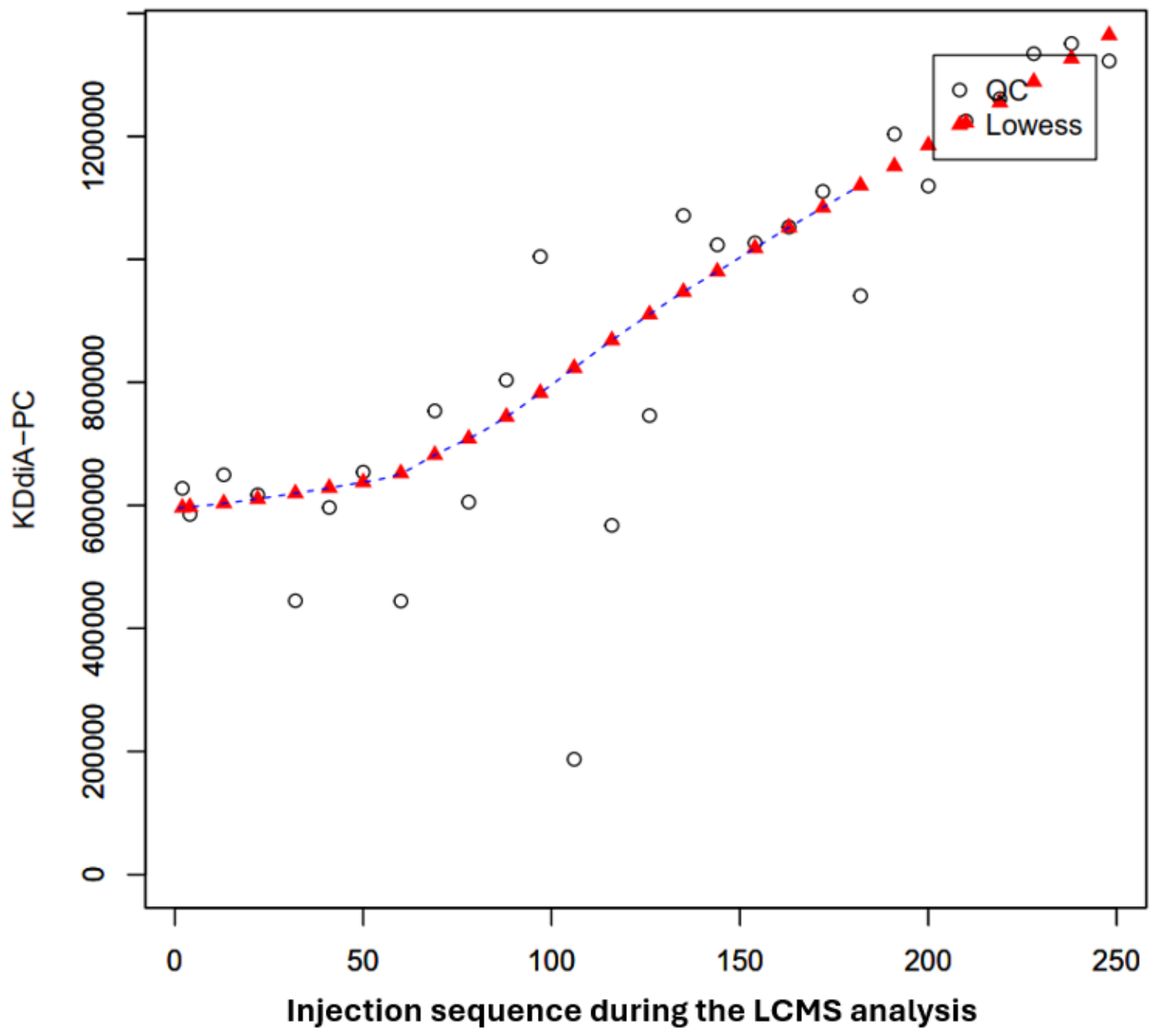

Figure S1. Example QC curve for the lipid feature KDdiA-PC, which passed the QC RSD threshold and was retained for further analysis.

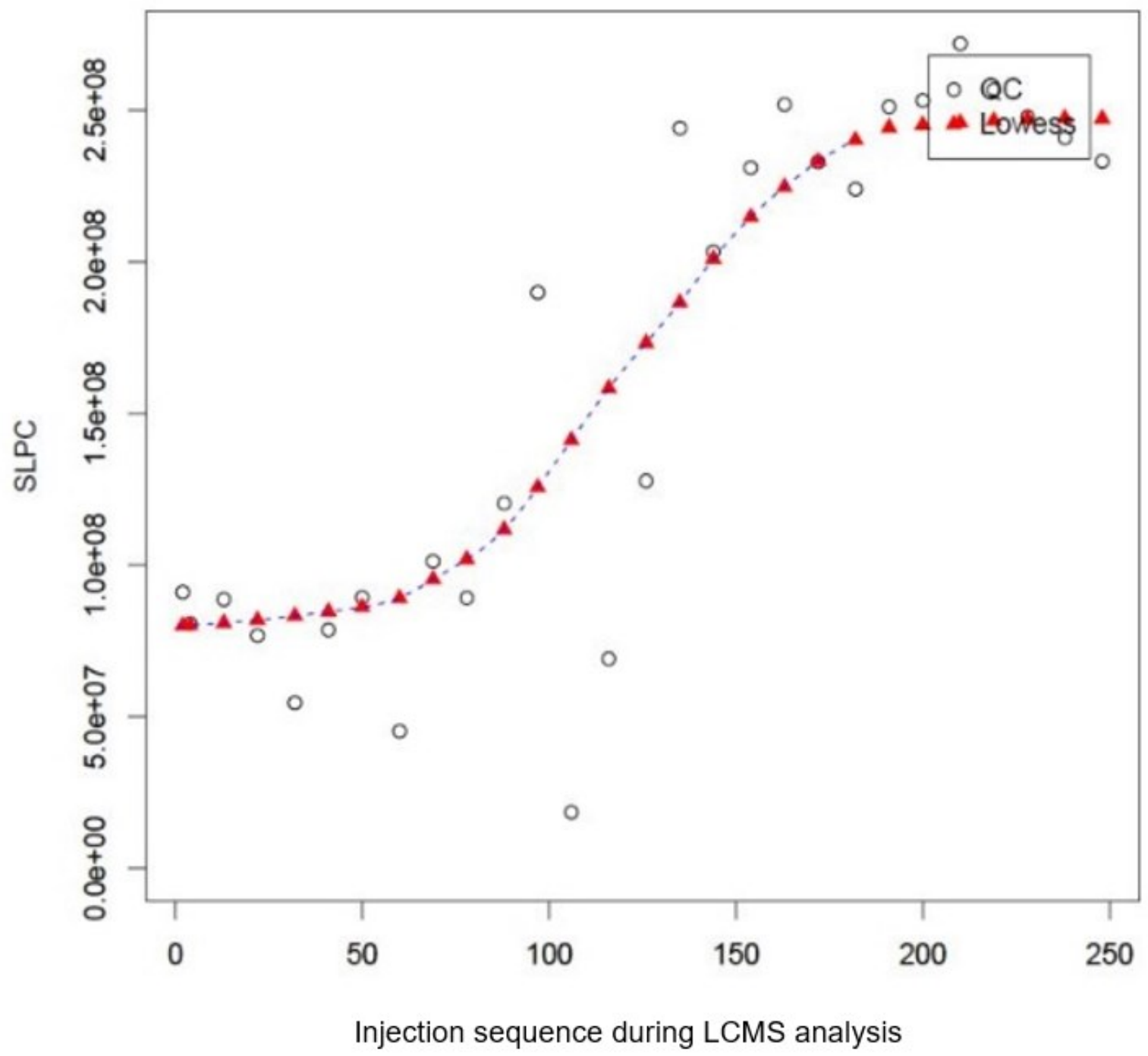

Figure S2. Example QC curve for the lipid feature SLPC, which exceeded the maximum QC RSD threshold of 30% and was therefore removed from the dataset and excluded from further analysis

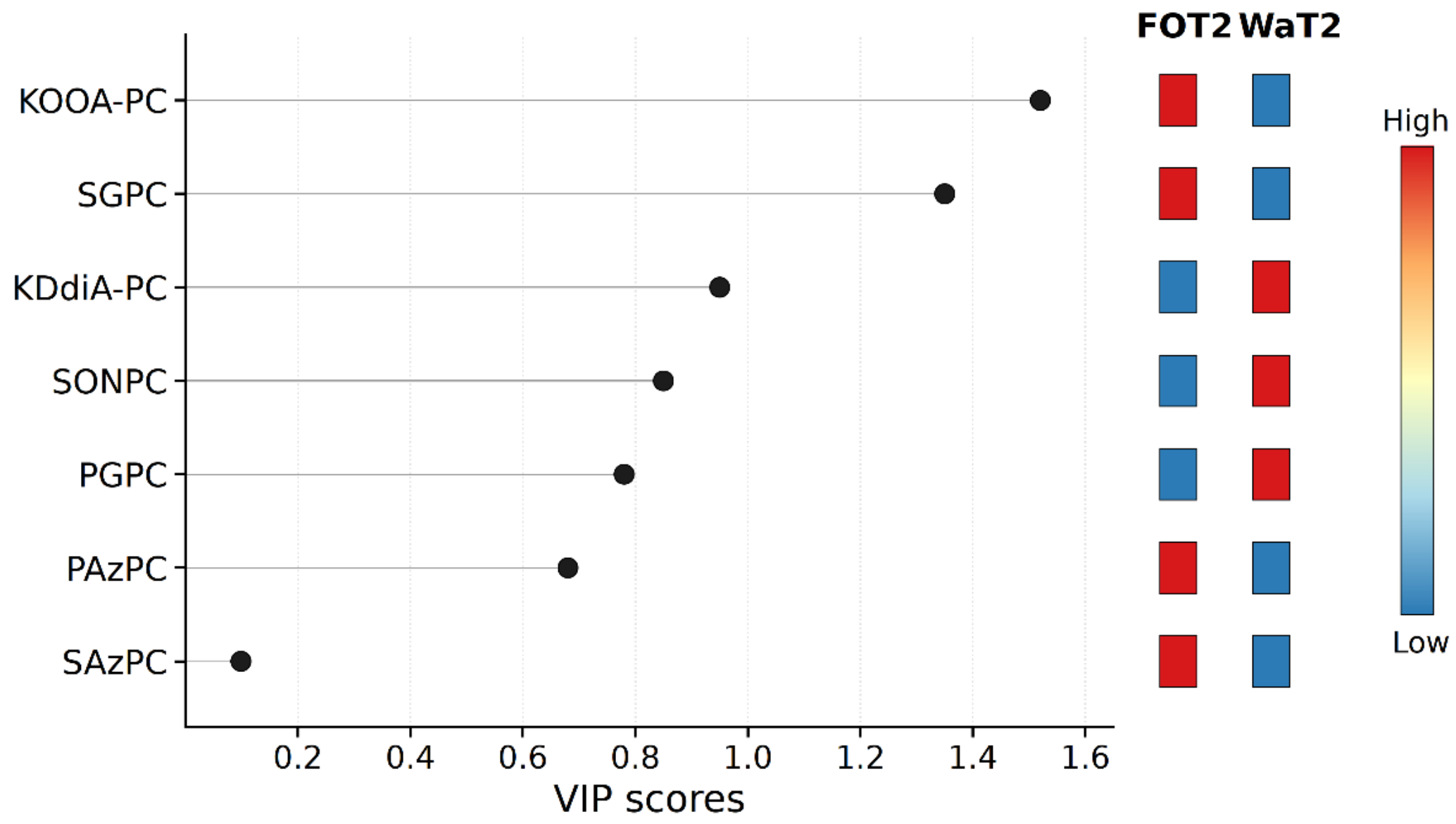

(a) Important features identified by PLS-DA at ΔT2

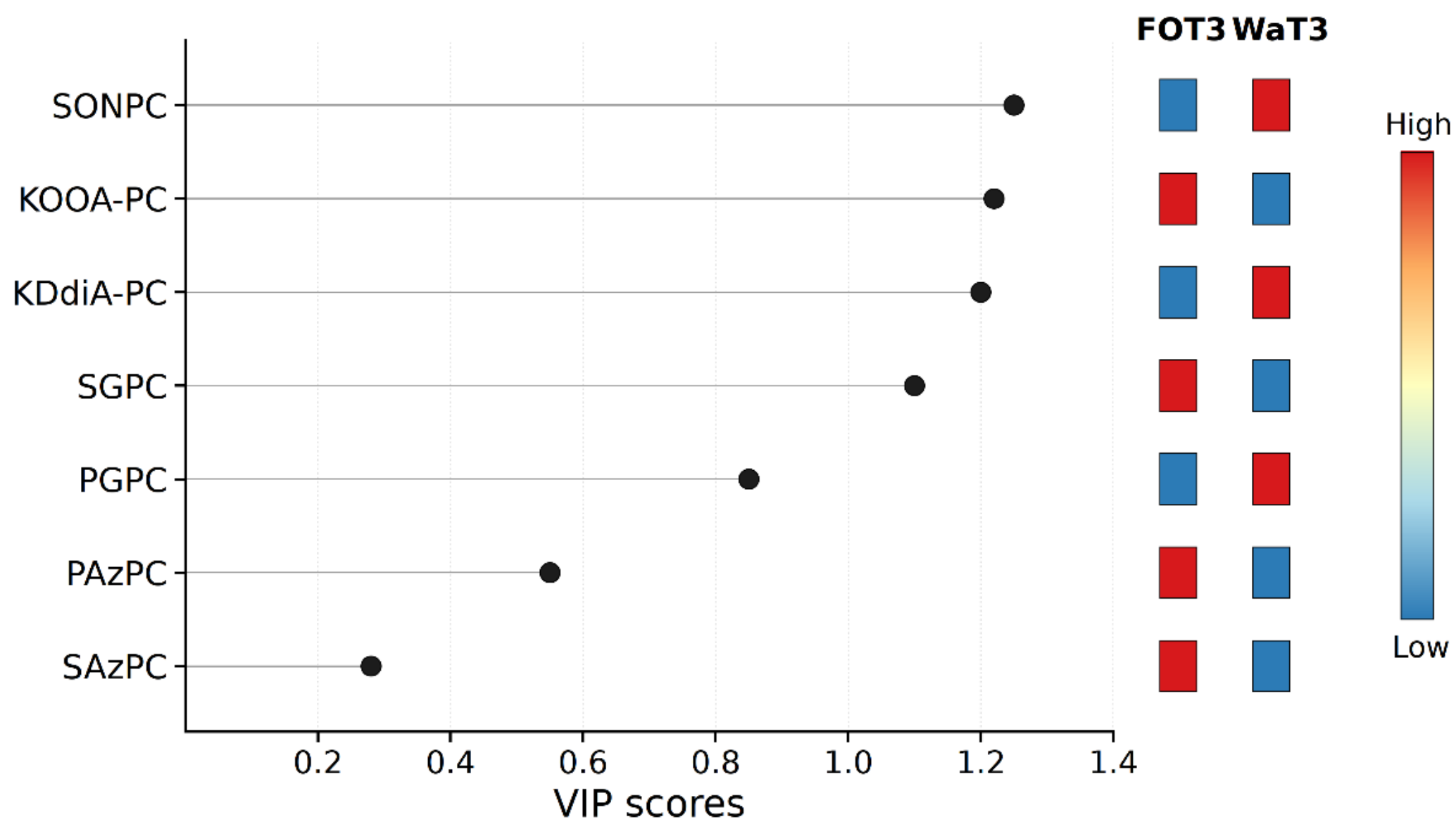

(b) Important features identified by PLS-DA at ΔT3

Figure S3. VIP scores from OPLS-DA model at (a) ΔT2 and (b) ΔT3, indicating SONPC, KOOA-PC, and KDdiA-PC as the dominant discriminatory features as expected due to their significance in expressing the oxidation level

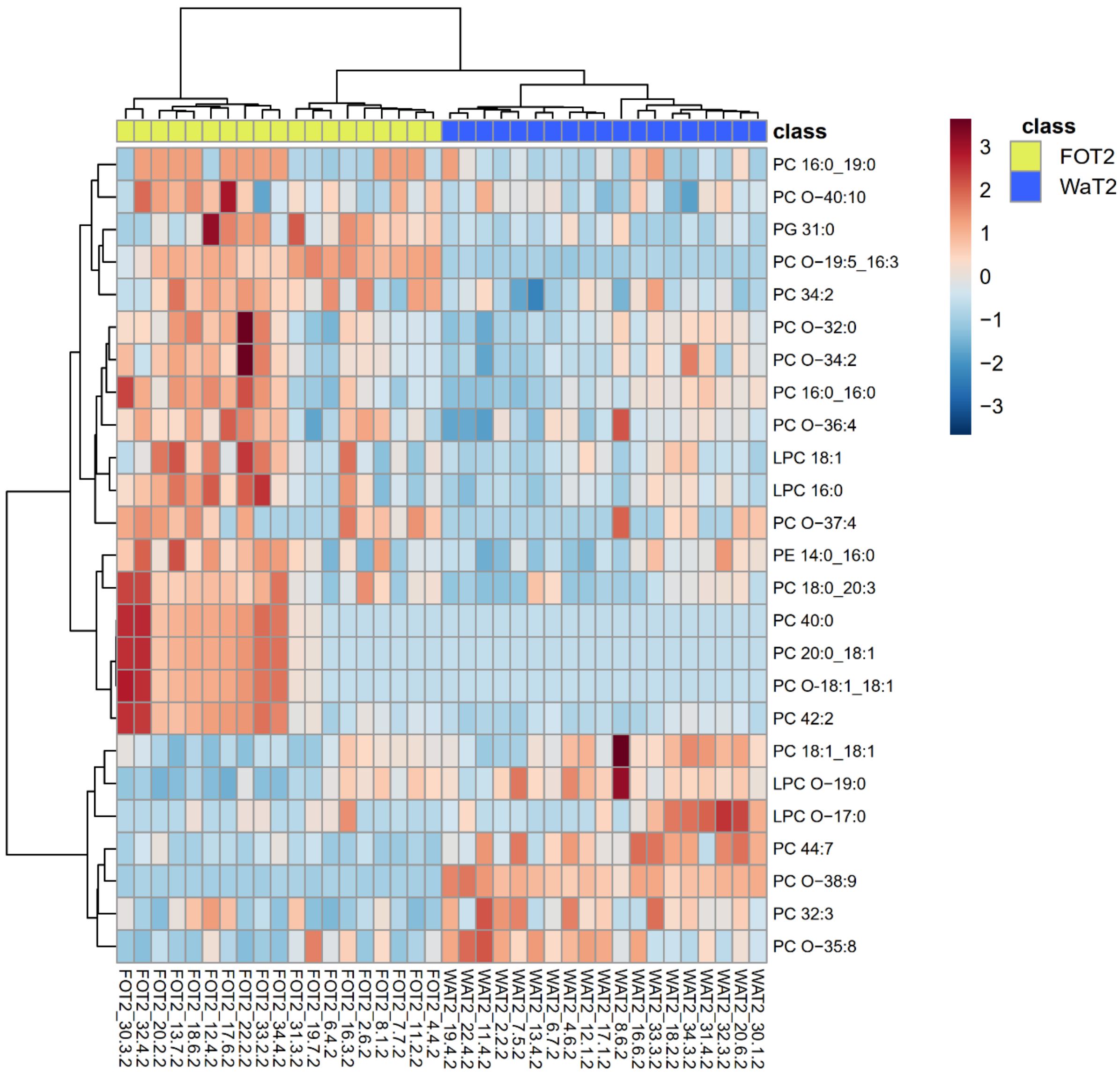

Figure S4. Heatmap showing the normalised peak intensity levels of individual lipid species across WaT and FOT from untargeted lipidomics at T2. Lipid species (rows) and biological replicates (bottom columns) were clustered based on Euclidean distance and Ward's method. The color scale represents the Z-score (normalized value), where red indicates higher relative abundance and blue indicates lower relative abundance within the same row. The top horizontal color bar identifies the two main class categories (FOT and WaT).

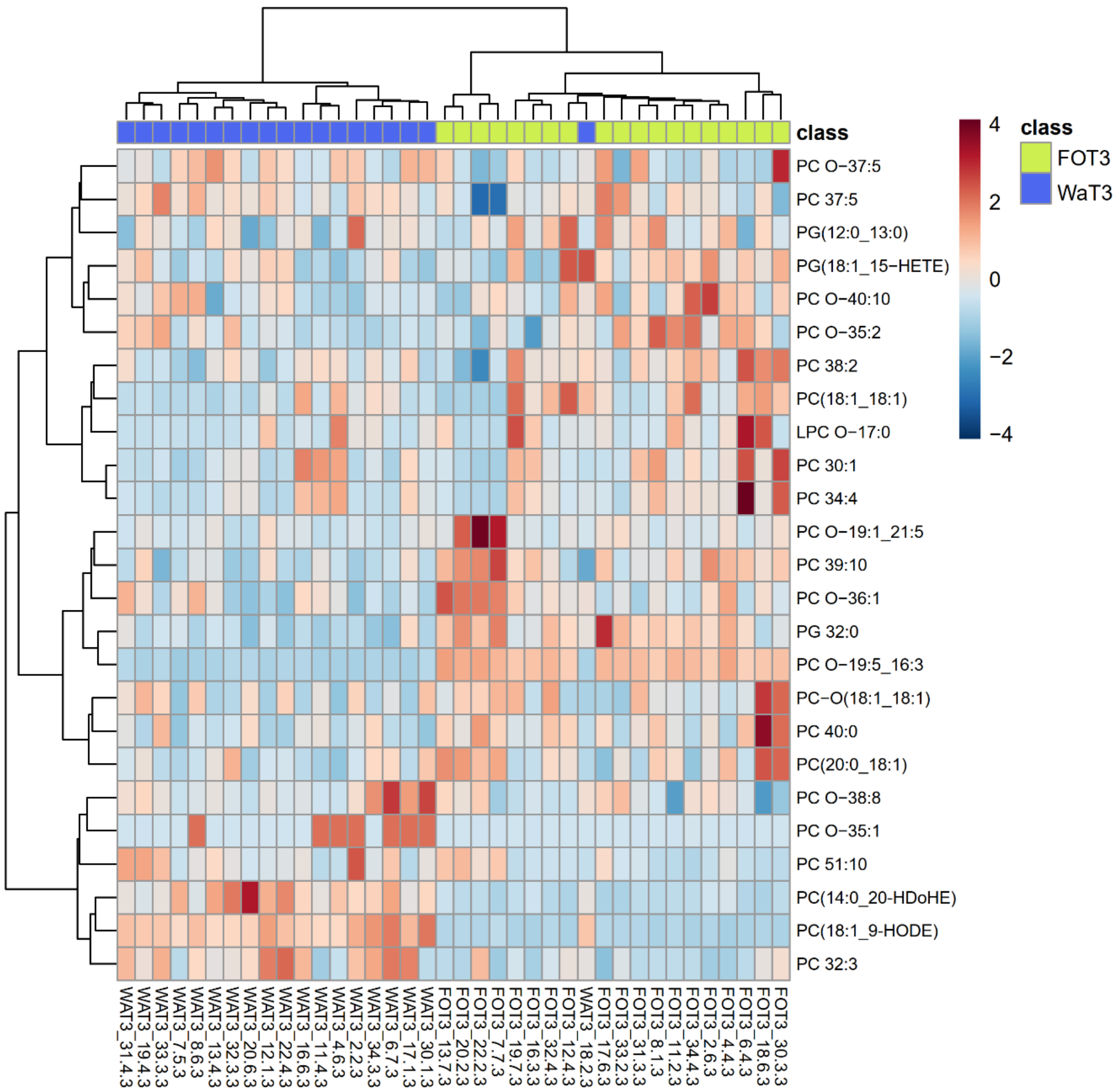

Figure S5. Heatmap showing the normalised peak intensity levels of individual lipid species across WAT and FOT from untargeted lipidomics at T3. Lipid species (rows) and biological replicates (bottom columns) were clustered based on Euclidean distance and Ward's method. The color scale represents the Z-score (normalized value), where red indicates higher relative abundance and blue indicates lower relative abundance within the row. The top horizontal color bar identifies the two main class categories (FOT and WaT).

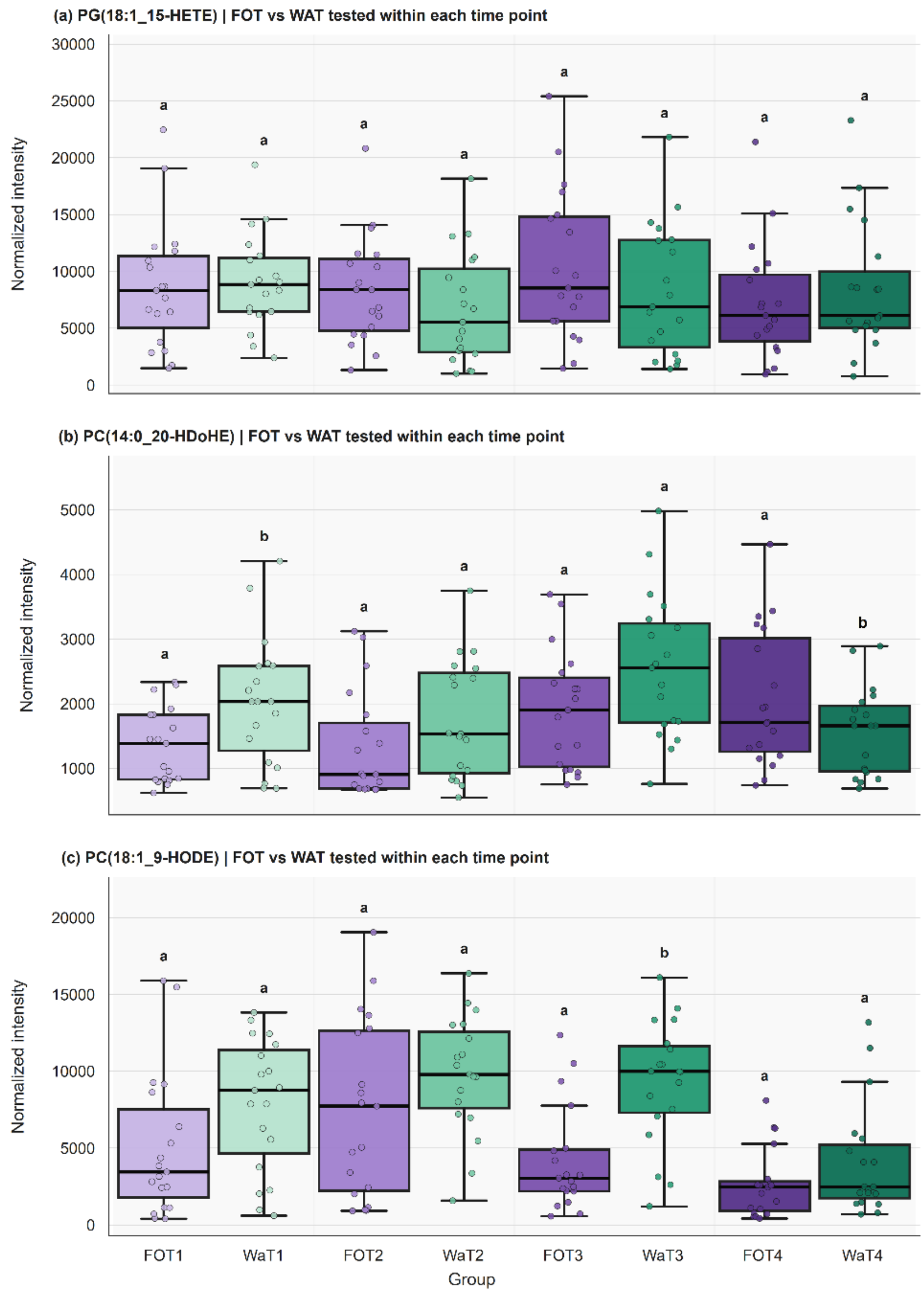

Figure S6. Boxplots showing normalized intensity of selected lipid metabolites across FOT and WAT groups at four time points. (a) PG(18:1_15-HETE), (b) PC(14:0_20-HDoHE), and (c) PC(18:1_9-HODE). Individual points represent samples; boxes show the interquartile range with median lines. FOT and WAT groups were compared within each matched time point using Tukey HSD. Different letters indicate significant differences between FOT and WAT at the same time point ($p < 0.05$); shared letters indicate no significant difference.

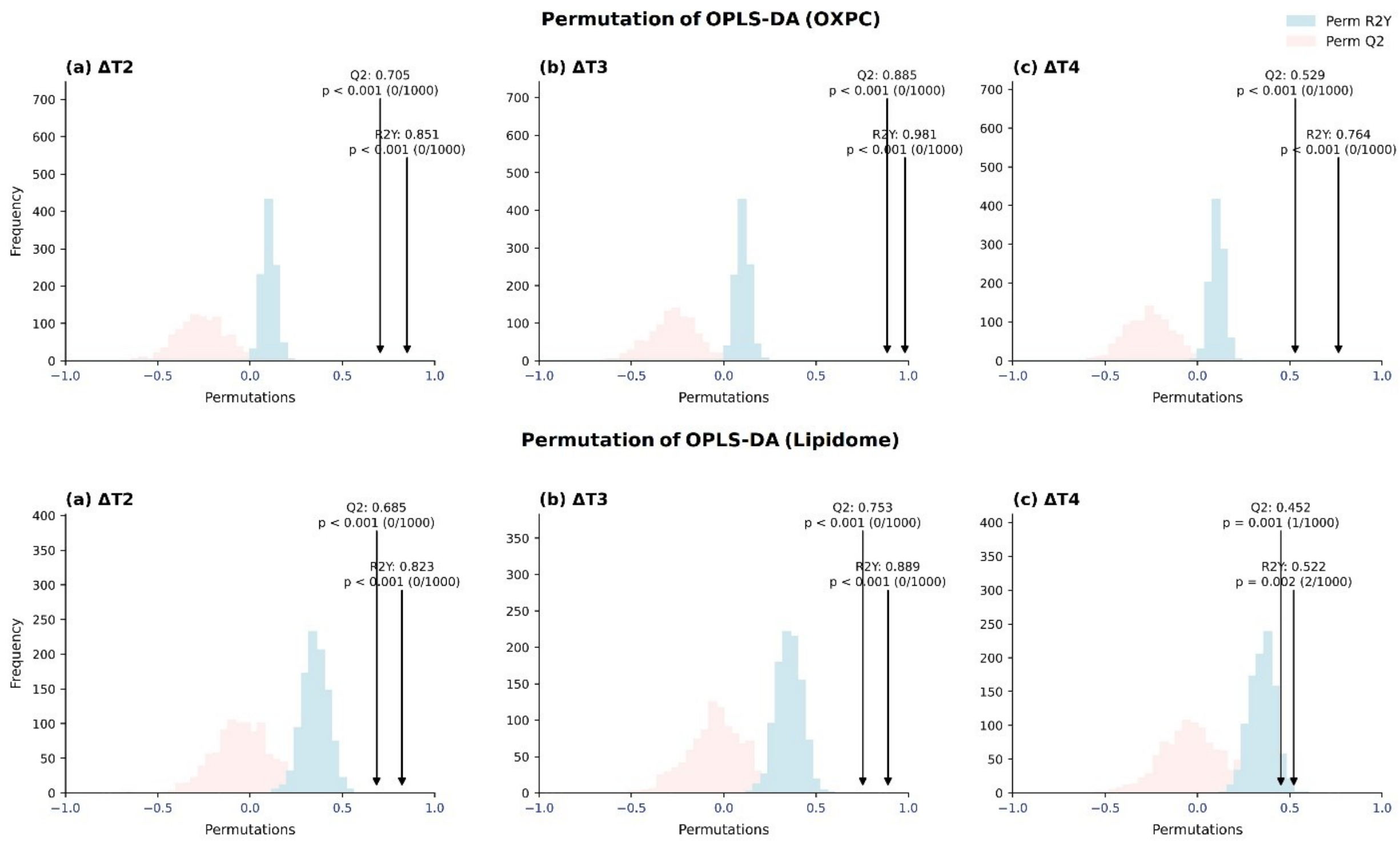

Figure S7. Panel plots of permutation test results for the OPLS-DA models shown in Figures 3 and 5. The first row corresponds to the oxPCs dataset, and the second row to the phospholipidome dataset: (a) ΔT2 (T2 – T1), (b) ΔT3 (T3 – T1), and (c) ΔT4 (T4 – T1). P-values were derived from 1000 random label permutations for each supervised OPLS-DA model. R^2Y and Q^2 values are annotated directly on the corresponding panels in Figures 3 and 5.

Supplementary Material 2

Created by

Contents of Report https://lipidomicstandards.org, version v2.6.1

Separation Workflow

Table S7 Overall study design
Table S8 Lipid extraction
Table S9 Analytical platform
Table S10 Quality control
Table S11 Method qualification and validation
Table S12 Reporting

Sample Descriptions

Table S13Plasma Oxidized Lipids/Human/Plasma

Separation Workflow

Table S7 Overall study design

Title of the study	Oxidomics of Human Plasma Reveals Lower Oxidized Lipids after Acute Intake of Polyphenol-Enriched Flaxseed Oil: A Randomized Crossover Exercise Trial		
Document creation date	06/01/2026	Principal investigator	Marc Pignitter
Institution	Department of Physiological Chemistry, University of Vienna	Corresponding Email	marc.pignitter@univie.ac.at
Is the workflow targeted or untargeted?	Targeted	Clinical	No

Table S8 Lipid extraction

Extraction method	2-phase system	pH adjustment	None
2-phase system	MTBE	Special conditions	ice-cold methanol containing 50 µg/mL butylated hydroxytoluene (BHT)
Were internal standards added prior extraction?	Yes		

Table S9 Analytical platform

Ionizaton additives	Ammonium acetate, Formic acid	Number of separation dimensions	One dimension
Separation type 1	LC	Separation mode 1 (liquid)	RP
Detector	Mass spectrometer	MS type	QQQ
MS vendor	Shimadzu	Ion source	ESI
MS Level	MS^2	Mass window for precursor ion isolation (in Da total isolation window)	1
Mass resolution for detected ion at MS^2	Low resolution	Resolution at MS^2	Unit
Recording mode of raw data at MS^2	Centroid mode	Was/Were additional dimension/techniques used	No

Table S10 Quality control

Blanks	Yes	Type of Blanks	Extraction blank, Solvent blank
Quality control	Yes	Type of QC sample	Sample pool

Table S11 Method qualification and validation

Method validation	Yes	Lipid recovery	Yes
Dynamic quantification range	Yes	Limit of quantitation (LOQ)/Limit of detection (LOD)	Yes
Precision	Yes	Accuracy	Yes
Guidelines followed	None		

Table S12 Reporting

Are reported raw data uploaded into repository?	Available on request	Are metadata available?	Available on request
Raw data upload	Available on request		

Sample Descriptions

Table S13 Plasma Oxidized Lipids / Human / Plasma

Storage and collection conditions	Available	Provided preanalytical information	Freeze-thaw cycles, Preservation method
Temperature handling original sample	4-8 °C	Instant sample preparation	No
Storage temperature	-80 °C	Freeze-thaw cycles	1
Additives	EDTA		

Supplementary Material 3

Created by

Contents of Report https://lipidomicstandards.org, version v2.6.1

Separation Workflow

Table S14 Overall study design
Table S15 Lipid extraction
Table S16 Analytical platform
Table S17 Quality control
Table S18 Method qualification and validation
Table S19 Reporting

Sample Descriptions

Table S20 Plasma Lipid/ Human/ Plasma

Separation Workflow

Table S14 Overall study design

Title of the study	Oxidomics of Human Plasma Reveals Lower Oxidized Lipids after Acute Intake of Polyphenol-Enriched Flaxseed Oil: A Randomized Crossover Exercise Trial		
Document creation date	06/01/2026	Principal investigator	Marc Pignitter
Institution	Department of Physiological Chemistry, University of Vienna	Corresponding Email	marc.pignitter@univie.ac.at
Is the workflow targeted or untargeted?	Untargeted	Clinical	No

Table S15 Lipid extraction

Extraction method	2-phase system	pH adjustment	None
2-phase system	MTBE	Special conditions	ice-cold methanol containing 50 µg/mL butylated hydroxytoluene (BHT)
Were internal standards added prior extraction?	Yes		

Table S16 Analytical platform

Ionizaton additives	Ammonium acetate, Formic acid	Number of separation dimensions	One dimension
Separation type 1	LC	Separation mode 1 (liquid)	RP
Detector	Mass spectrometer	MS type	QTOF
MS vendor	Agilent	Ion source	ESI
MS Level	MS^2	Mass window for precursor ion isolation (in Da total isolation window)	0.01
Mass resolution for detected ion at MS^2	High resolution	Resolution at m/z 200 at MS^2	20000
Mass accuracy in ppm at MS^2	5	Recording mode of raw data at MS^2	Profile mode
Was/Were additional dimension/techniques used	No		

Table S17 Quality control

Blanks	Yes	Type of Blanks	Extraction blank, Solvent blank
Quality control	Yes	Type of QC sample	Sample pool

Table S18 Method qualification and validation

Method validation	Yes	Lipid recovery	Yes
Dynamic quantification range	No	Limit of quantitation (LOQ)/Limit of detection (LOD)	No
Precision	Yes	Accuracy	Yes
Guidelines followed	None		

Table S19 Reporting

Are reported raw data uploaded into repository?	Available on request	Are metadata available?	Available on request
Raw data upload	Available on request	Additional comments	unprocessed dataset can be requested to the corresponding author upon reasonable justification

Sample Descriptions

Table S20 Plasma Lipid/ Human / Plasma

Storage and collection conditions	Available	Provided preanalytical information	Freeze-thaw cycles, Preservation method
Temperature handling original sample	4-8 °C	Instant sample preparation	No
Storage temperature	-80 °C	Freeze-thaw cycles	1
Additives	EDTA		
